# The *LuWD40-1* Gene Encoding WD Repeat Protein Regulates Growth and Pollen Viability in Flax (*Linum Usitatissimum* L.)

**DOI:** 10.1371/journal.pone.0069124

**Published:** 2013-07-30

**Authors:** Santosh Kumar, Mark C. Jordan, Raju Datla, Sylvie Cloutier

**Affiliations:** 1 Department of Plant Science, University of Manitoba, Winnipeg, Manitoba, Canada; 2 Cereal Research Centre, Agriculture and Agri-Food Canada, Winnipeg, Manitoba, Canada; 3 National Research Council, Saskatoon, Saskatchewan, Canada; United States Department of Agriculture, United States of America

## Abstract

As a crop, flax holds significant commercial value for its omega-3 rich oilseeds and stem fibres. Canada is the largest producer of linseed but there exists scope for significant yield improvements. Implementation of mechanisms such as male sterility can permit the development of hybrids to assist in achieving this goal. Temperature sensitive male sterility has been reported in flax but the leakiness of this system in field conditions limits the production of quality hybrid seeds. Here, we characterized a 2,588 bp transcript differentially expressed in male sterile lines of flax. The twelve intron gene predicted to encode a 368 amino acid protein has five WD40 repeats which, *in silico*, form a propeller structure with putative nucleic acid and histone binding capabilities. The LuWD40-1 protein localized to the nucleus and its expression increased during the transition and continued through the vegetative stages (seed, etiolated seedling, stem) while the transcript levels declined during reproductive development (ovary, anthers) and embryonic morphogenesis of male fertile plants. Knockout lines for *LuWD40-1* in flax failed to develop shoots while overexpression lines showed delayed growth phenotype and were male sterile. The non-viable flowers failed to open and the pollen grains from these flowers were empty. Three independent transgenic lines overexpressing the *LuWD40-1* gene had ∼80% non-viable pollen, reduced branching, delayed flowering and maturity compared to male fertile genotypes. The present study provides new insights into a male sterility mechanism present in flax.

## Introduction

Male sterility in flax (*Linum usitatisimmum* L.) was first documented in the early nineteen hundreds from *F_2_* of a cross between a blue flower oilseed type and a white flower fibre type where both parents were hermaphrodite with no male sterility [1]. The anthers in these male sterile *F_2_* lines sometimes developed to a stage where little viable pollen was produced suggesting an incomplete sterility mechanism. The male sterility phenotype was heritable because mostly male sterile plants gave rise to plants exhibiting the same phenotype. The sterile *F_2_* plants also had smaller petals that seldom opened. In a reciprocal cross with the blue flower oilseed type as the female parent, one out of four *F_2_* plants were male sterile [1].

In the present study we observed the occurrence of male sterility in an *F_2_* cross between oilseed type flax accession Double Low and cultivar AC McDuff. Both Double Low and AC McDuff are fertile parents. A gene with homology to EST LuP1225D10 (EB713752) encoding a tryptophan-aspartate (WD) repeat protein from AC McDuff was found to be expressed exclusively in temperature sensitive male sterile plants of flax [2]. Hence, we decided to characterize this gene in order to determine its role in male sterility.

WD proteins are present in bacteria such as *Thermomonospora curvata* [3] and *Cyanobacterium synechocystis* [4] but are more prevalent in eukaryotes. The earlier hypothesis suggested intragenic duplication and recombination events as the source of WD-repeats [5] but recent evidence hints at divergence of members of this protein family at different time points during evolution [6]. The WD proteins contain varying numbers of WD repeats and act as protein-protein and protein-nucleic acid interaction domain. The WD domain consists of 40 amino acids with a glycine-histidine (GH) di-peptide near the N-terminal, a conserved aspartic acid located before the WD repeats and the signature WD di-peptide at the C-terminal end [7], [8]. The first identified, and so far the best characterized WD protein, is the β-subunit of heterotrimeric G protein [9]–[11]. Based on crystal structure studies of G proteins, the WD proteins acquire a highly symmetrical β-propeller fold structure with each repeat containing a small four-stranded β sheet [10], [11]. A single β-propeller may contain 4 to 8 repeats but 7 or 8 repeat propellers are the most common [12]. The large β-propeller of WD40 proteins are composed of ∼300 amino acids which can interact through the top, the bottom and the circumference of the propeller [6]. Studies in yeast interactome demonstrated that WD40 proteins are involved in more protein-protein interactions than any other domains [13], [14]. The functional versatility of WD40 proteins is owed to their ability, (i) to target different substrates selectively similar to F-box proteins [15] (ii) to recruit different substrates in binding modes similar to the peptide-in-groove binding of clathrin [16] or by distinct binding modes through the top and side of the WD domain as in the G_β_ of the G proteins [11] (iii) to interact through insertion motifs as in MAD3 protein [17] or inter-blade binding grooves of WD40 domains for ligand binding as in Sro7 protein [18].

The WD40 proteins are involved in diverse cellular functions including cell division [19], cytoskeletal organization [20], [21], vesicle formation and trafficking [22] and transcriptional regulation [23], [24]. WD40 protein mediated transcriptional regulation by histone recognition [25] and arrangement of histone tails for the spreading of chromatin [26] impart silencing and thus has epigenetic consequences in regulating growth and development. WD40 protein malfunctions can lead to disease development [5]. Although much of the earlier evidence on the role of WD40 proteins came from animal studies, research in the model species Arabidopsis has advanced the understanding of their diverse functions in plants [27], [28]. Studies in Arabidopsis and rice revealed the presence of 85 and 78 WD40 proteins, respectively [29]. The majority of the WD repeat containing genes discovered have crucial functions in plants such as the F-box protein transport inhibitor response1 which is an auxin hormone receptor [30]. The *fertilization-independent endosperm* gene regulates flowering and seed dormancy [31]. The WD40 domain containing G protein coupled receptors play a vital role as hormone receptors and signalling components [32]. Cyclophilin71 interaction with histone H3 is essential for chromatin-based gene silencing affecting organogenesis [33]. A WD40 protein encoding gene, the promoter of which interacts with the dehydration response element, may be involved in stress tolerance in foxtail millet [34]. The Arabidopsis Yaozhe (YAO) is a nucleolar WD40 protein involved in embryogenesis and gametogenesis [35]. Rice Immature Pollen1 containing five WD40 repeats regulates late pollen development in rice [36]. *OsLIS-L1* gene encoding the Lissencephaly Type-1-Like protein is involved in plant height and male gametophyte formation in rice [37].

In this study, we report on the cloning and characterization of a novel WD40 protein encoding gene *LuWD40-1* corresponding to the EST LuP1225D10, a homologue of which was initially identified to be expressed exclusively in Chinese male sterile flax accession [2].

## Materials and Methods

### Gene sequence analysis

Clone LuP1225D10 (EB713752), obtained from a cDNA library constructed from 12 days after flowering flax bolls [38], was completely sequenced. Extended promoter and 5′UTR sequences were obtained from the assembly of AC McDuff short Illumina reads [39] mapped onto the whole genome shotgun (WGS) sequence of flax cv CDC Bethune (LinUsi v1.1, NCBI genome project #68161) [40]. The transcription start site (TSS) was identified using the bioinformatics pipeline for TSS signal analysis (http://fruitfly.org/seq_tools/promoter.html) [41]. Open reading frame (ORF) prediction was done using the ORF Finder tool from NCBI and the DNAMAN software (http://www.lynnon.com/). Promoter analysis was performed with PLAnt Cis-acting regulatory DNA Elements (PLACE) [42], PLANT Promoter Analysis Navigator (PlantPAN) and Weight Matrix patterns of PLant regulatory sequences (ScanWM-PL) available on the Softberry web portal (http://linux1.softberry.com/berry.phtml?topic=scanwmp&group=programs&subgroup=promoter). Sequence homologies were calculated with ClustalW2 [43] and a dendrogram was constructed using the neighbour-joining method implemented in the Molecular Evolutionary Genetics Analysis (MEGA) software version 5.1 [44].

### RNA-Seq and data normalization

Tissue samples of flax cv CDC Bethune were collected from embryos (globular, heart, torpedo, mature), seeds, etiolated seedlings, stems, ovaries and anthers. Total RNA was isolated from each of these tissue samples using RNeasy® Plant Mini Kit (Qiagen Inc., Mississauga, Ontario, Canada) to construct the nine tissue-specific libraries. RNA-Seq was performed on the Illumina platform (Genome Analyzer II) at the National Research Council (Saskatoon, SK, Canada). Paired-end tagged (PET) sequence reads were generated for each library using one sample per lane. The PET RNA sequences from each tissue were aligned to the WGS sequence of flax [40] using Tophat [45] with default settings. Transcript assembly and transcript abundance were analysed using the cuffdiff modules of the Cufflinks package with default settings [46], [47]. To calculate expression levels from read counts, the Cufflinks package was used to count the reads that map to a transcript and then to normalize the mapped read counts to the length of the transcript. In addition, to compare expression of transcripts across runs, the read counts were normalised for the total sequence yield of the machine [46]. These two normalization steps were implemented by calculating the Fragments Per Kilobase of transcripts per Million mapped reads (FPKM) [46] to reduce variability arising from assembly of PET reads, length of transcript, RNA composition and variations in library preparation. The normalized RNA-Seq dataset is available through the Total Utilization Flax GENomics (TUFGEN) project website (www.linum.ca) and can be downloaded from http://linum.ca/downloads/RNAseq.

### Constructs for plant transformation

Cloning was carried out using the Gateway system (Invitrogen, Carlsbad, CA, USA). Forward (5′-ATGCGCATGGACGCGACGAAC-3′) and reverse primers with sequences encoding the human influenza Hemagglutinin tag (5′-TCAAGCGTAGTCTGGGACGTCGTATGGGTACCCCCGGCTGCAGCG-3′) were designed to amplify the 1,107 bp full length cDNA of the gene. An additional reverse primer R_1_ (5′-CATATGCTGGTCCGTCATGGC-3′) was designed at the 5′end spanning introns and exons to distinguish the amplification product arising from cDNA or genomic DNA templates and was used in combination with the forward primer to amplify a 274 bp fragment (Fig. S1A) for generating the RNAi construct, for gene amplification in confirmation of transgenic lines and for semi-quantitative RT-PCR experiments. The full length gene without a stop codon was amplified using the same forward primer and the following reverse primer (5′-CCCCCGGCTGCAGCGGAAT-3′) and cloned into the CD3-685 vector (pEarleyGate103, ABRC) containing a GFP tag at the C-terminal end. Cloning and shuttling were performed as per manufacturer's instructions (Gateway® Cloning Technology, Invitrogen, Carlsbad, CA, USA). The PCR amplified gene fragments were cloned into pDONR221 entry clone and positive transformants were sequenced to confirm their accuracy. The gene constructs were then incorporated into pIPKb004 binary destination vector for overexpression (OE) and pIPKb009 for RNAi based knockout (KO) constructs [48]. The transgenic flax lines were tested for the presence of the transgene using a CaMV 35S promoter forward primer (5′-GATGACGCACAATCCCACTATCCT-3′) [48] and the gene specific internal reverse primer R_1_. The following forward (5′-TAGAGCTGACCAGGACAAACA-3′) and reverse (5′-GTTTATGAATGCGCTTGTCTCA-3′) primers were used to amplify the house-keeping gene adenine phosphoribosyl-transferase1 (*apt1*) [49] that served as the internal control for semi-quantitative RT-PCR [50].

### Transformation

Flax cultivar Prairie Grande was transformed according to the protocol of Wijayanto and McHughen [51] with the following modifications. The sterilized seeds were grown in the dark on Murashige and Skoog (MS) medium with 2% sucrose and 0.4% phytagel (MS2P). Sets of thirty hypocotyl segments per plate in multiple replicates were maintained at 24°C under a 16 h light and 8 h dark cycle for 4 days before bombardment. Plasmid DNA (5μl at 1μg/μl) was precipitated on sterilized 0.6 micron gold particles using CaCl_2_ and protamine as previously described [52]. The particle bombardment of 5μg plasmid DNA, per plate at 650 psi pressure and a distance of 6cm was done using a biolistic PDS-1000/He delivery system (Bio-Rad Laboratories, Mississauga, Ontario, Canada). The bombarded hypocotyls were maintained for an additional 4 days in the same conditions as the pre-culture before transferring them to MS2P medium, containing 5 mg/l hygromycin for 4 weeks followed by subculture with 20 mg/l hygromycin for 2–3 months until regeneration of shoots. Regenerating shoots were excised and transferred to Magenta jars containing 5 mg/l hygromycin in MS2P medium. The shoots that survived and formed roots were transferred to soil and grown in growth cabinets at 18°C under a 16 h light and 8 h dark cycle. The putative transformed flax plants were allowed to mature, flower, self-pollinate and set seeds.

For subcellular localization studies, onion epidermal cells were transformed with the GFP-tagged *LuWD40-1* gene using the same particle bombardment conditions as for the flax hypocotyls. The transfected epidermal layers were incubated in the dark for 48 h before microscopy.

### PCR and semi-quantitative RT-PCR

DNA was extracted from 10 mg lyophilised leaf tissue of the untransformed Prairie Grande and the transgenic lines using the QiagenDNeasy 96 plant kit (Qiagen Sciences, Maryland, USA). RNeasy® Plant Mini Kit (Qiagen Inc., Mississauga, Ontario, Canada) was used for RNA extraction from leaf tissue of the same genotypes. Total RNA was treated with TURBO DNA-*free*DNase (Ambion, Austin, Texas, USA) and cDNA synthesis was performed using Superscript™ II reverse transcriptase (Invitrogen, Carlsbad, California, USA) followed by RNase H (Invitrogen, Carlsbad, California, USA) treatment. The cDNA was quantified using Quanti-iT™ Ribogreen RNA reagent kit (Molecular probes, Eugene, Oregon, USA) [53]. Approximately 30 ng of genomic DNA extracted from leaf tissues of the transgenic lines was used as template to confirm the presence of the transgene construct. Semi-quantitative RT-PCR was performed with 4 ng cDNA from the leaf tissue of transgenic lines using the forward and R_1_ primers described above. PCR of DNA and cDNA samples were performed with melting temperature set to 62°C for 36 cycles and 28 cycles for PCR and semi-quantitative RT-PCR, respectively. Semi-quantitative RT-PCR is based on the relative expression measured during the linear phase of the reaction [54]. We performed amplification of the *LuWD40-1* target and the *apt1* control at 28 and 31 cycles and compared the intensity ratios by densitometry measurement using the AlphaImagerHP software (version 3.4, proteinsimple, Santa Clara, CA, USA). At 28 cycles, variations in gene to control ratios were obtained between the untransformed Prairie Grande and the transgenic lines, however, at 31 cycles, the gene to control ratios suggested near saturation of amplification (Fig. S2). The semi-quantitative RT-PCR experiment was repeated three times at 28 cycles. All protocols were performed according to the manufacturer's instructions.

### Microscopy

The flax pollen cells from the untransformed Prairie Grande and the transgenic lines stained with 2,5-diphenyl tetrazolium bromide (MTT) (Sigma, Oakville, Ontario, Canada) [55] were counted at 50× magnification on a Wild Heerbrugg M8 stereo microscope (Wild Heerbrugg, Heerbrugg, Switzerland) equipped with a fiber optic light source and illuminator cables. The transformed onion epidermal cells expressing GFP were visualized through a Zeiss AxioScope.A1 microscope (Carl Zeiss, Oberkochen, Germany) using EC Plan-NEOFLUAR lenses under 20× and 40× magnifications. The images were captured through an AxioCam lCc 1 camera mounted on the microscope and the Axio Vision software (Release 4.8.2) was used to process the images.

## Results

### Sequence analysis of *LuWD40-1*

A study on dominant genic male sterile flax identified a transcript that had 89% sequence identity with LuP1225D10 and was expressed exclusively in male sterile lines of Chinese flax accession [2]. The EST clone LuP1225D10 was generated from AC McDuff which is a fertile plant. However, when AC McDuff is crossed to another fertile parent, Double Low, the progeny shows male sterility which does not follow Mendelian inheritance. Here, we presented the molecular and functional characterization of the gene.

Sequencing of the cDNA clone LuP1225D10 generated a fragment of 1,638 bp including an 84 bp 5′ UTR, a 1,107 bp ORF and a 447 bp 3′UTR ending in a polyA tail. This sequence was submitted to Genbank as *LuWD40-1* (KC686833). The 5′UTR region extended using the AC McDuff Illumina short reads [39] confirmed that LuP1225D10 contained the full length ORF of a WD40 gene. The promoter sequences of CDC Bethune and AC McDuff were identical but the coding sequence of the CDC Bethune homologue *Lus10011938* was predicted to be 6 bp shorter than that of LuP1225D10. The *Lus10011938* sequence was 2,588 bp long with a 1,101 bp predicted ORF. This shorter predicted ORF for CDC Bethune was the result of an error in the prediction of the true start codon of the *ab initio* prediction and RNA-Seq data (Datla, unpublished) confirmed that the AC McDuff and CDC Bethune ORFs were both 1,107 bp in length. The 5′UTR of *LuWD40-1* was 1,034 bp with a TATA box located 30 bp upstream of the TSS. The complete transcript including the extended 5′ UTR sequence of *LuWD40-1* (2,588 bp) with its deduced amino acid sequence is shown (Fig. S1A). The genomic clone corresponding to the ORF of *LuWD40-1* from the flax WGS sequence is 4,471 bp and is composed of thirteen exons and twelve introns (Fig. S1B & S1C).

**Figure 1:**
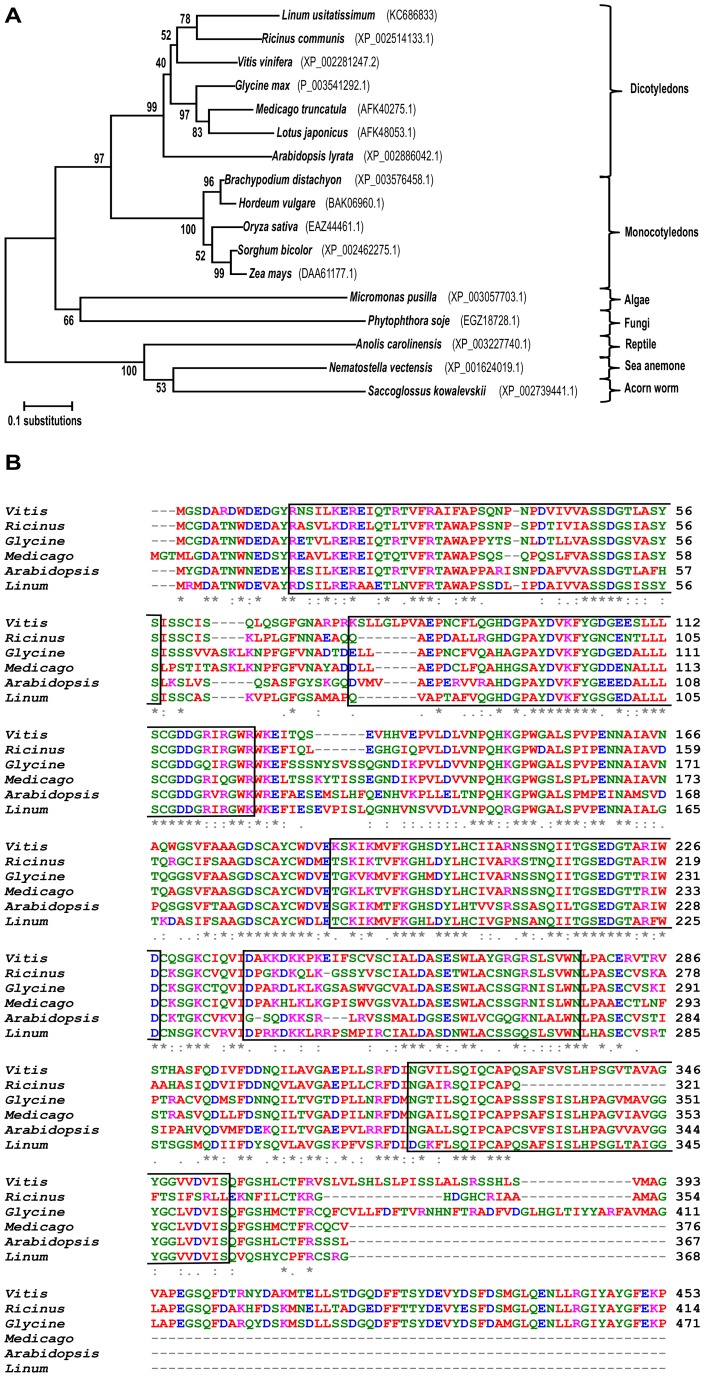
Phylogenetic and structural analysis of WD40 proteins. (A) An amino acid sequence based phylogenetic tree of WD40 proteins from various organisms. The phylogenetic tree was generated by the MEGA 5 software using the neighbour-joining algorithm, and bootstrap values based on 1000 replications are represented at the branch points. The relative amount of change is represented by the scale bar. (B) Deduced amino acid sequence alignment of WD40 repeat proteins from dicot species was generated by ClustalW2. WD40 repeats are shown in boxes. Sequences from other species that extended beyond *Linum* sequence at the 3`end and did not contain any WD40 domains were trimmed.

The *LuWD40-1* gene encodes a protein of 368 amino acids with a theoretical isoelectric point (pI) of 5.66 and an estimated average molecular mass of 39.7 kDa (Compute pI/Mw tool, www.expasy.org). A BLASTP search of the deduced amino acid sequence against the non-redundant protein database of NCBI showed high similarity to WD40 proteins such as the putative dead box ATP-dependent RNA helicase from *Ricinus communis* (E-value 4e-158; XP_002514133), the *Arabidopsis lyrata* transducin family protein (XP_002886042.1), DWA-1 like protein from *Cucumis sativus* and the *Arabidopsis thaliana* THO complex subunit 6 (NP_849989.1) proteins. Various unnamed proteins with WD40 domains but unknown functions from *Vitis* (CBI27884.3), *Glycine* (ACU18349.1) and *Medicago* (AFK40275.1) showed similarity ranging between 60–65%. The BLAST results also included putative orthologs from algae, fungi, reptiles and worms, all of which had WD40 domains. A protein sequence based phylogenetic tree showed clear divergence between WD40 proteins from the plant kingdom compared to species from other kingdoms (Figure 1A). Within plants, monocot and dicot WD40 proteins displayed evolutionary divergence. The flax WD40 protein was most closely related to the protein from castor bean (Figure 1A). A Simple Modular Architecture Research Tool (SMART) analysis of the LuWD40-1 encoded protein (http://smart.embl-heidelberg.de/) identified five WD40 domains of 38 to 43 amino acids residues in the dicot orthologs (Figure 1B). Variation in the protein sequence and indels were present, even within the WD40 domains (Figure 1B). The *lotus japonicus* sequence (AFK40275.1) was not included in the alignment because it was partial.

**Figure 2:**
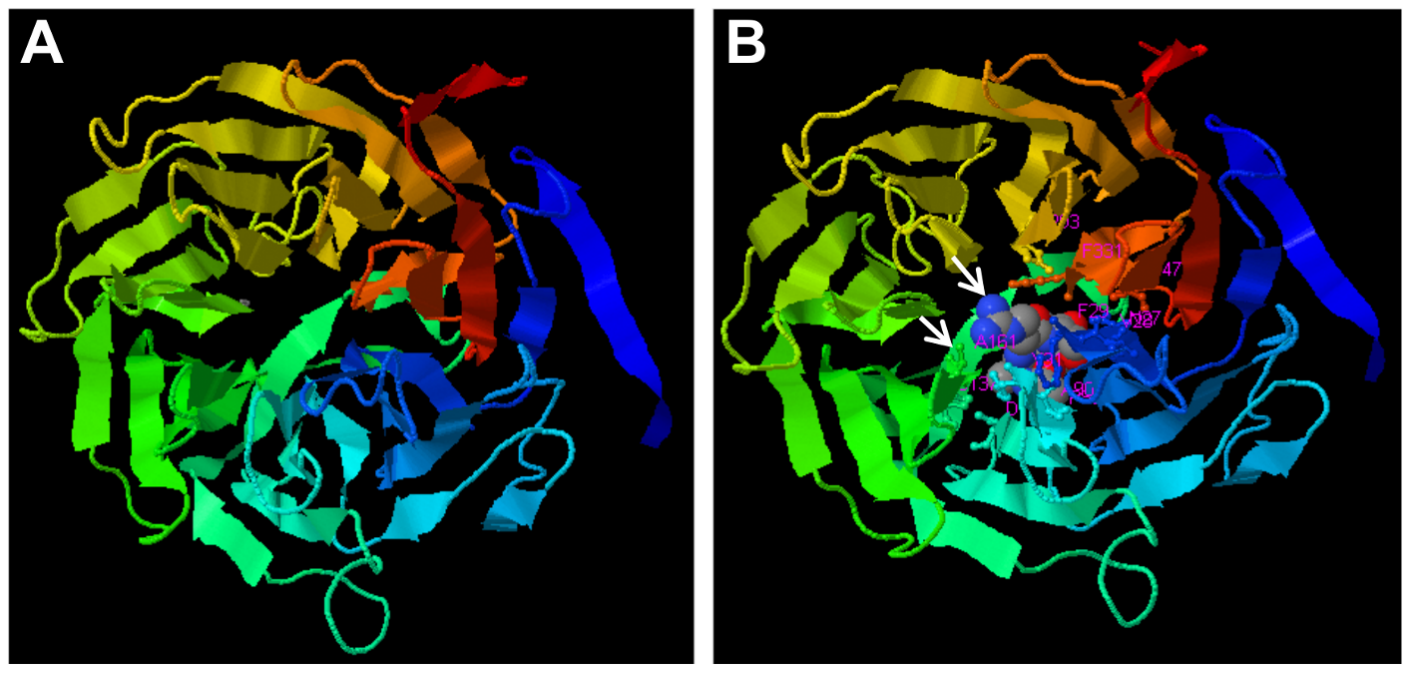
Structure of LuWD40-1 based on I-Tasser prediction. (A) Predicted propeller structure of LuWD40-1 protein based on 10 highly similar templates. The beta sheets and turns are drawn as arrows and lines, respectively. (B) Predicted binding sites on LuWD40-1 based on six most probable templates are shown with arrows. The LuWD40-1 protein is depicted as a cartoon and the binding proteins are illustrated as spheres.

An *ab initio* analysis of LuWD40-1 secondary structure was performed on the I-TASSER server [56]. The model with the highest C-score (−0.78) is shown (Figure 2A). The model was confirmed through the SWISS-MODEL workspace [57] with a QMEAN Z-Score of −5.89. LuWD40-1 with five WD40 repeats formed a seven blade propeller structure similar to proteins with seven WD repeats. Other WD40 domain containing proteins of the I-TASSER database also possessed nucleotide or histone binding characteristics such as the transcriptional repressor TUP1 [58] and the histone binding pRB associated protein p46 (RBBP7) [59] (Figure 2B). LuWD40-1 is predicted to have a histone binding site identical to the histone binding protein RBBP7.

### *In silico* promoter analysis and transcript profile of *LuWD40-1* in flax tissues

**Figure 3:**
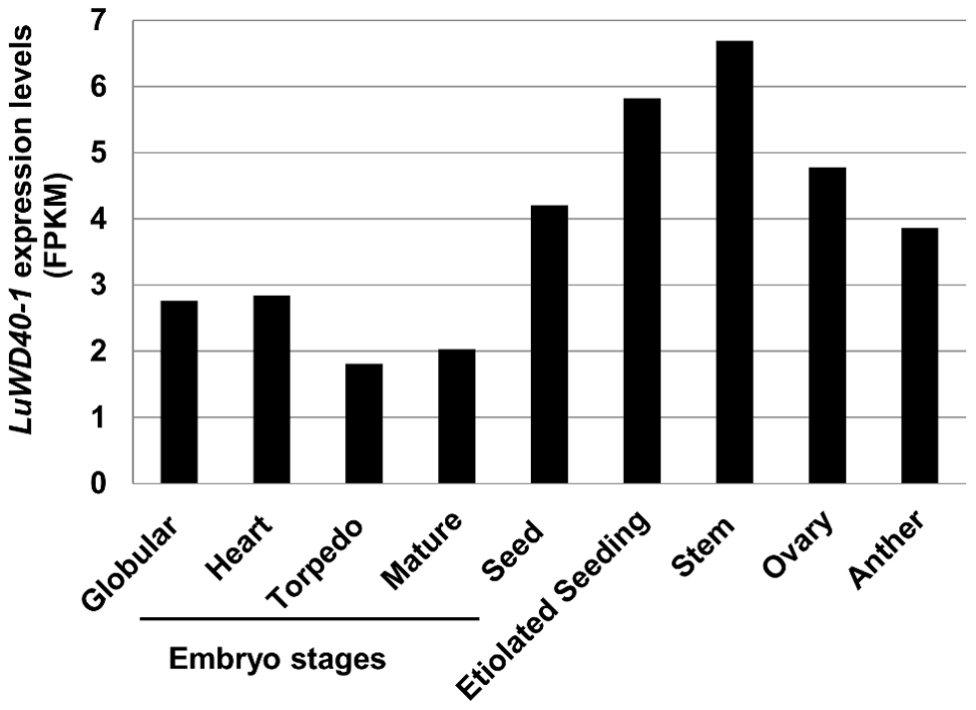
Transcript profile of *Lus10011938/LuWD40-1* based on RNA-Seq data. The RNA extracted from wild type plants were sequenced from various tissues. The abundance of *Lus10011938/LuWD40-1* transcripts in different tissue samples are represented by normalized FPKM values. RNA-Seq and related data normalization is described in the Materials and Methods section.

The *LuWD40-1* sequence of AC McDuff is identical to the predicted gene *Lus10011938* from CDC Bethune. A 1,500 bp sequence upstream of the TSS of *LuWD40-1* and *Lus10011938* was extracted from the assembly of AC McDuff and CDC Bethune genome sequences and signal scan searches were performed. The list of reported signals is compiled (Table S1). Signal sequences for induction of gene expression in stems and roots and repression in leaves (E2FCONSENSUS) were identified. Several elements for light and stress responsive activation of genes were also present. Multiple copies of GTGANTG10 and POLLEN1LELAT52 known to regulate pollen specific expression were detected. The tissue specific transcript profiling based on normalised number of transcripts from tissue specific RNA-Seq data showed results consistent with the predicted promoter signal scan results (Figure 3). The *Lus10011938/LuWD40-1* transcripts had relatively lower expression at all stages of embryo development but were abundant in seed, etiolated seedling, stem, ovary and anther tissues.

### Cellular localization of LuWD40-1 in onion epidermal cells

**Figure 4:**
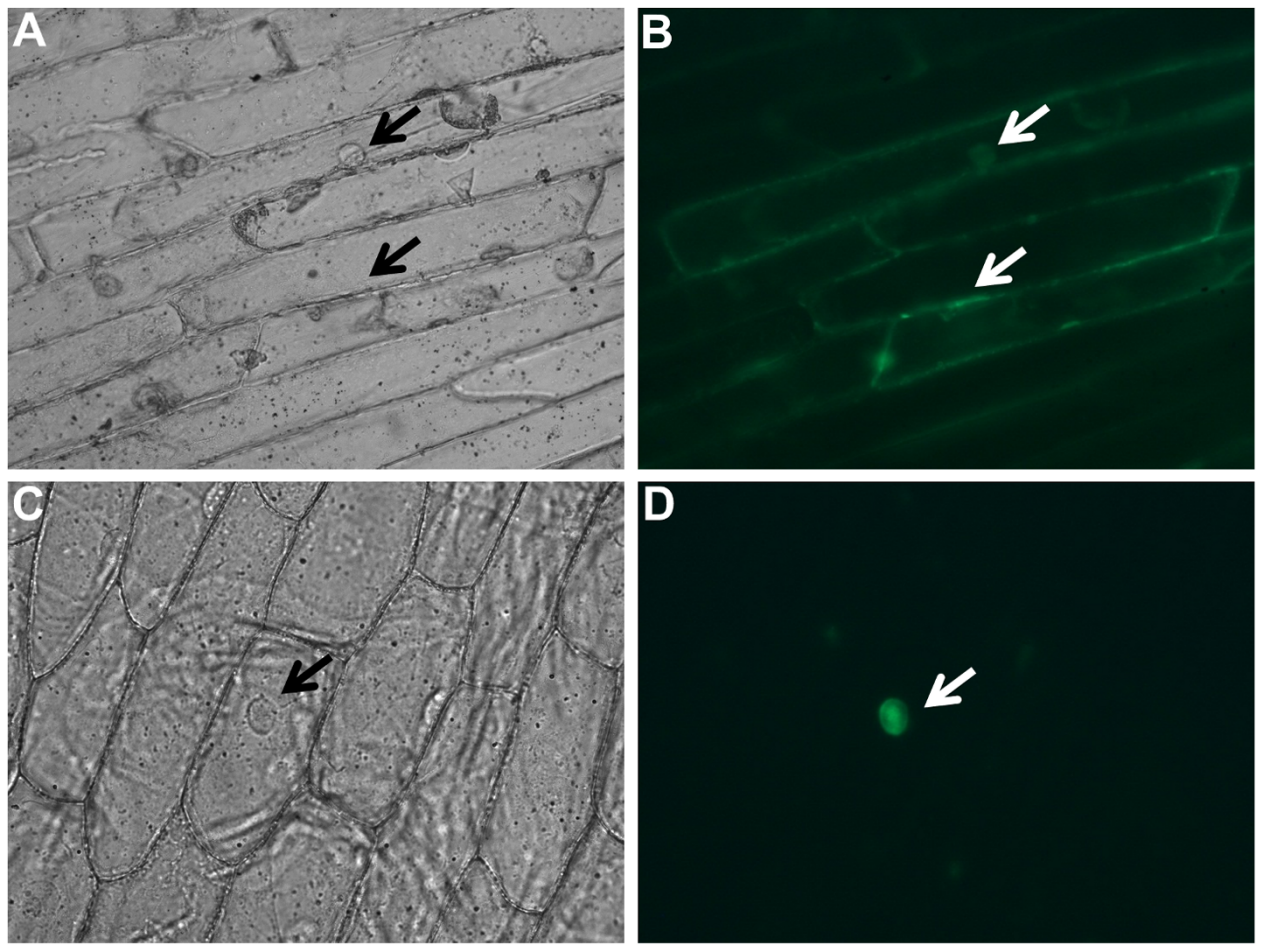
Subcellular localization of LuWD40-1-GFP in onion epidermal cells. The CaMV 35S promoter driven empty vector CD3-685-GFP and the CD3-685-LuWD40-1-GFP construct were transformed into onion epidermal cells using particle bombardment. The subcellular compartmentalization of the empty vector constructs under bright field (A) and fluorescence (B) shows diffusion throughout the cells (arrows). Transient expression of the LuWD40-1:GFP construct under bright field (C) and fluorescence (D) indicates localization in the nucleus (arrows).

The LuWD40-1 sequence was analysed through Subnuclear Compartments Prediction System (http://array.bioengr.uic.edu/subnuclear.htm) which suggested its localization in nuclear lamina. To confirm this prediction, onion epidermal cells were transformed with a *LuWD40-1-GFP* construct and an empty vector-GFP construct as control. Diffused green fluorescence signal was throughout the cells bombarded with the empty vector-GFP construct (Figure 4 A–B) but the GFP signal was localised exclusively in the nucleus in cells transiently expressing the *LuWD40-1-GFP* construct (Figure 4C–D).

### Phenotypic analysis of *LuWD40-1* transformed lines in flax

**Figure 5:**
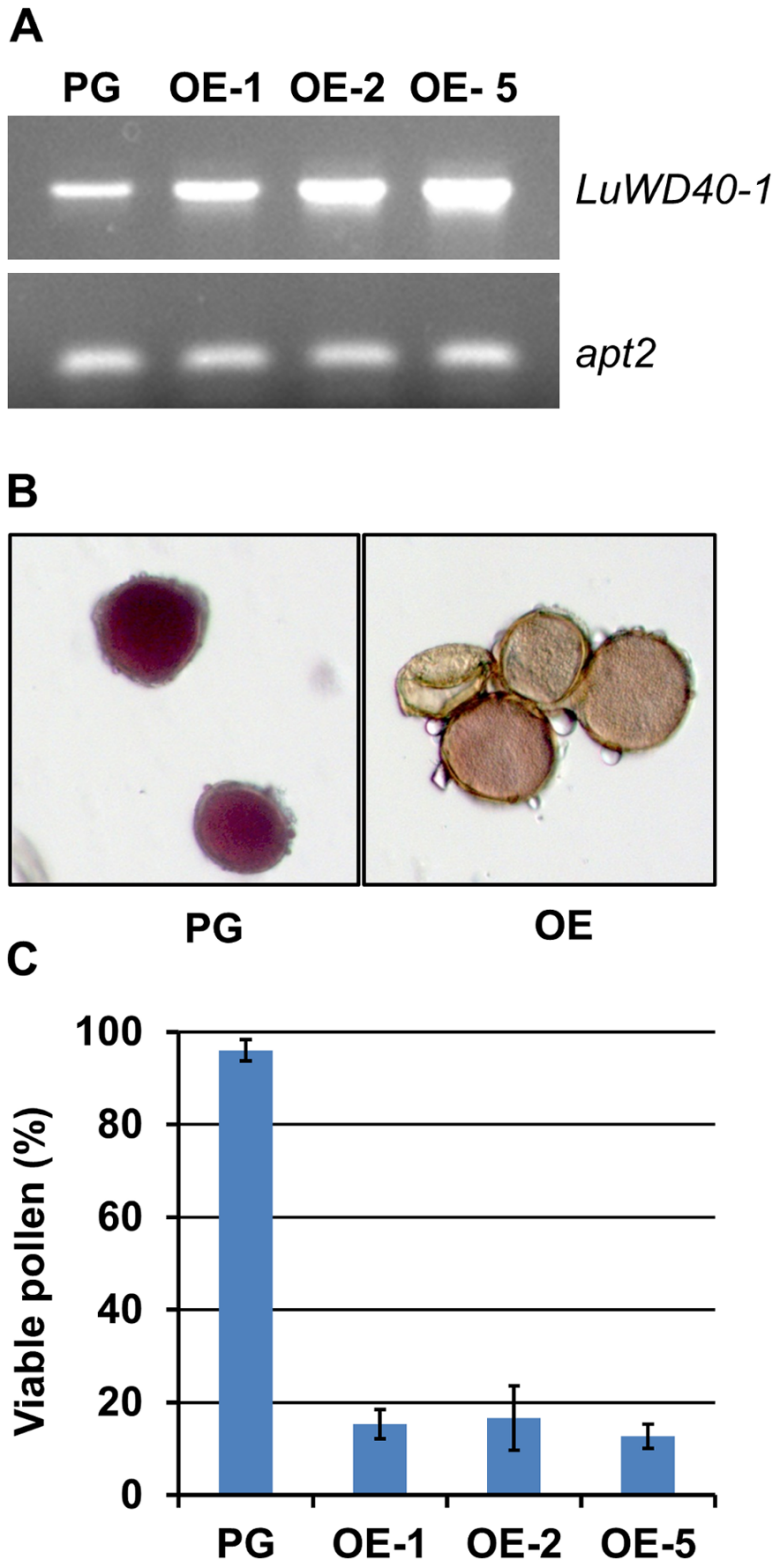
Transgenic lines overexpress *LuWD40-1* and show pollen sterility. (A) Semi-quantitative RT-PCR of *LuWD40-1* and the *apt1* control shows overexpression of *LuWD40-1* in the three T_2_ transgenic lines compared to the untransformed Prairie Grande (PG). (B) The PG lines with viable pollen (left) and the OE lines with non-viable pollen (right) as visualized by MTT staining for pollen viability. (C) Pollen viability in PG and OE lines. Pollen viability was measured using pollen grains from three flowers per plant.

The flax cultivar Prairie Grande was transformed with *LuWD40-1* constructs to develop OE and KO lines. A total of 1,770 and 2,940 hypocotyls were shot with the *LuWD40-1* OE and RNAi based KO constructs, respectively. None of the calli transformed with the *LuWD40-1* KO construct produced shoots, even after ten rounds of selection on hygromycin. Three out of the four T_1_ *LuWD40-1* OE lines that produced shoots were positive for presence of the transgene (Fig. S3) and were propagated through selfing to produce T_2_ lines. The OE T_1_ lines had low pollen viability and it was difficult to obtain viable seeds from these lines for further propagation. Only 2–5 viable seeds were obtained from each OE line and which were then grown to produce the T_2_ generation. The three T_2_ lines OE-1, −2 and −5 all showed overexpression of *LuWD40-1* as compared to the untransformed Prairie Grande (Figure 5A).

Pollen viability was estimated by the MTT test where viable pollen cells stain dark purple due to the conversion of the yellow MTT by the mitochondrial succinate dehydrogenase into a purple formazan product [60] while the non-viable pollen remains colourless (Figure 5B). The Prairie Grande pollen was dense and sank to the bottom of the tube upon immersion in water. The pollen grains of the OE lines mostly floated on the water surface. When visualized under the microscope with light source below the tissue, the Prairie Grande pollen was visualized as dark bodies due to the absorbance of the light by the active tissue within the cells whereas the OE pollen formed clear circular structures through which light was transmitted (Figure 5B). Pollen viability was estimated to be 96% in Prairie Grande but ranged from 12% to 16% in the T_2_ *LuWD40-1* OE lines (Figure 5C). Attempts were made to test the viability of the female reproductive organs in the OE lines. Cross pollination of 30 flowers in the three OE lines with Prairie Grande pollen resulted in seed set in ten OE flowers (∼33% success) suggesting retention of female fertility.

**Figure 6:**
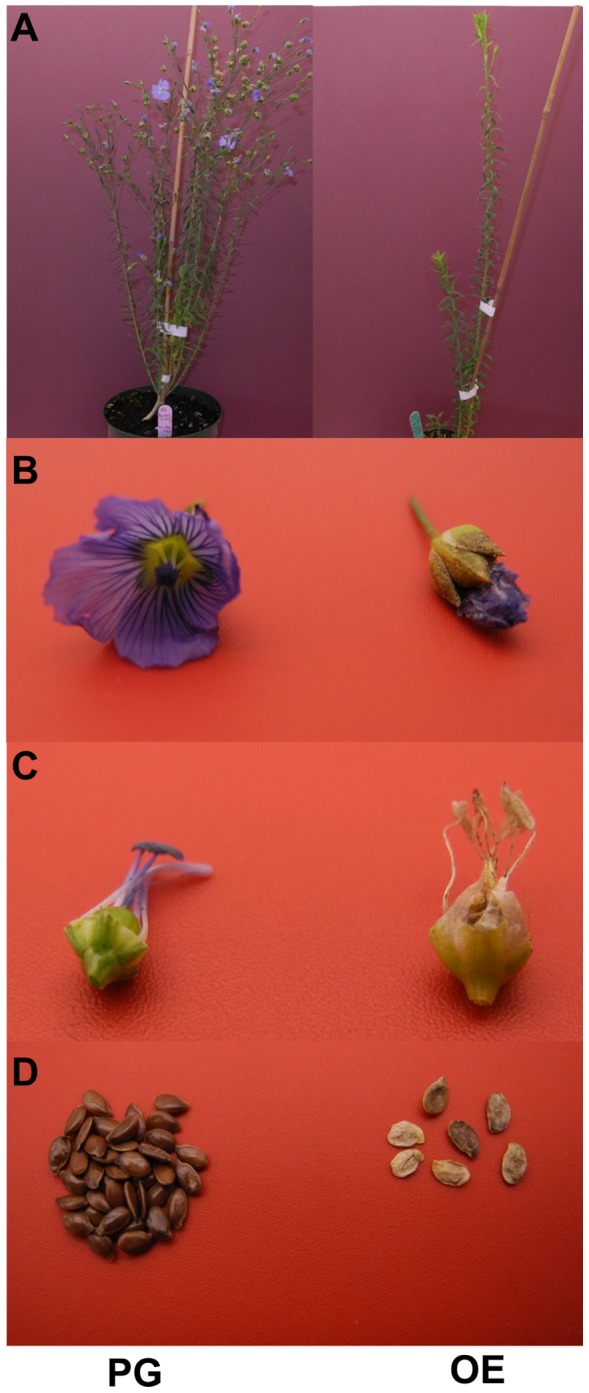
Phenotypic characterization of *LuWD40-1* transgenic lines. **Images comparing the phenotypes of Prairie Grande (PG) and OE lines for (A) whole plant architecture, (B) flower morphology, (C) reproductive structures and (D) seed quality.**

The male sterility observed in the *LuWD40-1* OE lines limited our ability to test sufficient numbers of individuals for each transgenic line generated. Hence, the physiological differences were compared between Prairie Grande and the three transgenic OE lines and not within each lineage. The OE lines showed clear signs of delayed growth. The slower growth habit of OE plants translated into significantly delayed flowering phenotype that and took ∼80 days to flower compared to Prairie Grande which reached that stage in ∼57 days (Fig. S4A). Fewer adventitious buds led to the production of fewer branches in OE lines compared to Prairie Grande which produced ∼10 branches per plant whereas the OE lines only had three (Fig. S4B). With fewer branches, the OE plants grew taller averaging 70 cm whereas Prairie Grande averaged 50 cm (Figure 6A; Fig. S4C). The petals of the Prairie Grande flowers open completely and fell prior to boll development (Figure 6B) while flowers of the OE lines remained closed and the petals persisted in that shape until maturity. Removal of the petals revealed normal development of reproductive organs in Prairie Grande but in OE lines, the anthers were shrivelled and had empty pollen cells (Figures 5B and 6C). Numerous plump dark brown seeds developed on Prairie Grande whereas the seed set and quality was severely impeded in OE lines (Figure 6D). The majority of the seeds that developed on OE lines were shrivelled, bleached and had poor germination.

## Discussion

Flax is a dual purpose crop grown for its omega-3 fatty acid rich seed oil (linseed) and its stem fibre (fibre flax). The increasing importance and value of the crop have justified the development of genetic resources to assist plant breeding efforts directed towards higher yielding varieties. The current advances include the first flax genome sequence [40], a collection of expressed sequence tags (ESTs) from more than 10 different tissues [61], a consensus genetic map [62], a physical map [63] and thousands of SNP markers [39].

Linseed and fibre types belong to the same species, *Linum usitatissimum*, a diploid self-pollinated crop with an estimated genome size of ∼375 Mb and 15 chromosomes (2n  = 2×  = 30). In flax, self-pollination occurs just prior to flower petal opening. The flower only lasts part of a day during which non-fertilized ovaries are still receptive, providing an opportunity for cross-pollination, although limited because pollen flow and insect-mediated pollination are minimal in flax [64]. Except for the recent development of a hybrid in China [65], flax varieties around the world are pure lines. Three independent and essential elements require investigations to justify the establishment of flax as a hybrid crop: (1) the quantification of its heterotic potential, (2) the development of an efficient male sterility system and (3) the establishment of a cost effective and reliable hybrid seed production system. Here, we investigated the *LuWD40-1* gene in flax for its potential use in a flax male sterility system.

The *LuWD40-1* homologue was found to be expressed exclusively in male sterile lines of Chinese flax accession tested only at the flower bud stage [2]. In our study, however, the *LuWD40-1* transcripts were present in all tested stages of CDC Bethune (Figure 3) as well as developing bolls of AC McDuff (EST Lu1225D10), both being male fertile flax accessions. The overexpression of *LuWD40-1* in male fertile accession Prairie Grande caused male sterility.

Analysis of the transcript features showed the presence of a large 5′UTR (Fig. S1A) possibly due to the retention of an intron during alternative splicing upstream of the ATG start codon. Suggestive evidence of intron selection comes from large scale analysis of human and mouse annotated genes where intron retention in UTR regions was demonstrated in ∼15% and 22% of transcripts, respectively [66], [67]. Large 5′UTRs are characteristics of regulatory genes encoding growth factors, homeodomain proteins and various receptors involved in post-transcriptional modifications [68]. They are also known to affect the expression pattern of their genes. In Arabidopsis, the long intron in the 5′UTR of elongation factor 1α-A3 enhances its gene expression in a length dependent manner [69].

The WD40 protein has putative orthologs in worms and reptiles, as well as lower and higher plants (Figure 1A), however, considerable differences exist between the protein sequences, even within dicot species (Figure 1B). Variations within the WD40 motifs account for their functional versatility as the most active protein interactor. Similar to the well characterized transducin family with seven WD repeats [11], LuWD40-1is also predicted to form a seven blade propeller structure even though it harbours only five WD repeats (Figure 2A). Although uncommon, other WD40 proteins with five WD repeats also form seven bladed propeller structures such as the coronin family of proteins [70].

The localization of LuWD40-1 into the nucleus (Figure 4) is consistent and supports the *in silico* prediction for interaction with histone binding proteins (Figure 2B). Several WD40 proteins such as WDR5, NURF55 and RbBP4/7 are classified as chromatin readers because they can recruit chromatin modifiers to specific sites on the chromosome by direct interaction with histones [71]. In addition to protein-protein interactions, WD40 repeat proteins such as BUB3 and Rae1 also act as docking proteins for assembly of protein complexes that regulate mitotic checkpoints [72], [73]. The Arabidopsis WD40 repeat protein NEDD1 regulates microtubule development during cell proliferation [74]. A recent report identified the WD40 protein interaction with nucleic acid where a non-coding RNA transcript from the HOXA locus interacted directly with WDR5 [75]. These diverse WD40 proteins and their nucleic acid binding characteristics make them key regulatory candidates in transcriptional and post-transcriptional regulation during gametogenesis and growth.

The *Lus10011938/LuWD40-1* transcripts were expressed in all analysed developmental stages of flax (Figure 3). The *Lus10011938/LuWD40-1* transcript was highly expressed in stems (Figure 3) and the fact that the KO lines failed to produce shoots from calli suggests a putative essential role in shoot formation as also found with WD40 protein mutants that showed reduced shoot growth resulting in shorter plants [37]. The flax lines overexpressing *LuWD40-1* had delayed flowering, severely reduced pollen viability, branching and growth (Figure 5 and Fig. S4). These phenotypes are likely the result of the constitutive overexpression including ectopic missexpression of the *LuWD40-1*. The presence of promoter elements such as GTGANTG10 and POLLEN1LELAT52 that cause anther specific expression [76] is consistent with transcript abundance of *Lus10011938/LuWD40-1* in anthers (Figure 3), however, constitutive overexpression of the same gene affects male gametophyte development leading to the production of sterile pollen. Imbalance in overall transcript profile could result in improper protein interactions of LuWD40-1 protein in pollen tissues. This hypothesis is supported by studies where pollen and anthers showed expression of approximately 15,000 genes, all of which were highly regulated [77].

Near complete male sterility (∼90% non-viable pollen) in a population arising from an *F_2_* cross between oilseed type flax accession Double Low and cultivar AC McDuff was found in approximately 4% of the lines while 8% were partially sterile (∼50% non-viable pollen). The occurrence of male sterility in the *F_2_* generation did not follow any simple ratios of gene inheritance or interaction. Epigenetic mechanisms involving small RNAs and/or transposable elements (TE) which are highly expressed in pollen vegetative nucleus cells but not in female gametophytes must be investigated [78].

Although other WD40 proteins like YAO and OsLIS-L1 have been implicated in embryo development, plant height and pollen formation [35], [37], they differ considerably from LuWD40-1 by their additional functional motifs. The Arabidopsis YAO, with its nuclear localization signal followed by seven WD40 repeats, is expressed ubiquitously in all plant tissues [35].The rice OsLIS-L1 contains a LisH motif, a CTLH domain and nine WD40 repeats, out of which, four form the G_β_ homology domain [37] indispensable for inducing male sterility. No other domains but the WD40 repeats could be identified in *LuWD40-1*. Moreover, we observed the phenotypes (Figure 6A–D) in OE lines whereas our knockout lines did not produce shoots, suggesting conserved functions of different WD40 proteins in diverse pathways of plant morphogenesis and reproduction.

To explore the possibility of using *LuWD40-1* OE lines to develop hybrids, we crossed the male sterile flowers of OE lines with pollen from Prairie Grande. A total of ten out of 30 cross-pollinated flowers set seeds suggesting the fertile state of ovaries. The low rate of cross-pollination efficiency could be due to pollen incompatibility mechanisms. Constitutive expression of the *LuWD40-1* gene may also interfere with cellular processes affecting pollen tube growth in the style of OE lines similar to the Pollen tube Defective WD40 (PD40) gene in tobacco [79]. More likely, it reflects the inherent difficulties associated with controlled crossing such as ovary receptivity and pollen developmental stage at the time of pollination.

The current study lays a foundation for the generation of male sterile flax lines providing basic physiological characterization of *LuWD40-1*. More insights can be gained by studying the pollen specific expression of this gene in flax to separate the negative effects on plant growth from the desired male sterility phenotype. It is imperative to understand the inheritance of this system for its implementation in the field.

## Supporting Information

Figure S1 **Sequence and schematic representation of the LuWD40-1 protein encoded by cDNA clone LuP1225D10.** (A) Nucleotide and deduced amino acid sequence of LuWD40-1 with 5′ and 3′ untranslated regions. The second in frame ATG selected in the *ab initio* annotation of *Lus10011938* is italicized. The 5′ region amplified for semi-quantitative RT-PCR experiment is underlined (B) Schematic representation of the genomic clone corresponding to *LuWD40-1* ORF drawn to scale. The boxes represent exons and lines represent introns. (C) DNA sequence of the genomic clone representing the open reading frame of *LuWD40-1.*
(PDF)

Figure S2 **Semi-quantitative RT-PCR. Amplification of the target gene** ***LuWD40-1*** **and the control gene** ***apt1*** **from the three transgenic lines overexpressing** ***LuWD40-1*** **and the untransformed Prairie Grande shows that the reactions had not reached saturation at 28 cycles but that saturation was achieved at 31 cycles.** Ratios (*LuWD40-1*:*apt1*) at the bottom were calculated by measuring the amplicon intensity by densitometry using the AlphaImagerHP software version 3.4 (proteinsimple, Santa Clara, CA, USA). Note: the Gateway recombination tags in the *LuWD40-1* primers increase the amplification size to 332 bp (274+29 bp tags in each forward and reverse primers). Molecular marker (M) is the 1KB Plus DNA ladder (Invitrogen, Carlsbad, California, USA).
(PDF)

Figure S3 **Confirmation of T_1_ transgenic lines by PCR using CaMV35S and gene specific primer combination on genomic DNA extracted from leaves.** Details of primers and PCR conditions are available in the Materials and Methods section.
(PDF)

Figure S4 **Physiological characterization of three** ***LuWD40-1*** **transgenic lines.** Graphs showing differences between Prairie Grande (PG) and overexpressing (OE) lines in physiological parameters: (A) bolting time, (B) branching and (C) height of the plants.
(PDF)

Table S1 **Transcription factor binding sites in the putative** ***LuWD40-1*** **promoter.**
(PDF)
