# Supplementary material for: The LuWD40-1 Gene Encoding WD Repeat Protein Regulates Growth and Pollen Viability in Flax (Linum Usitatissimum L.)
Source: PLoS One. 2013 Jul 30;8(7):e69124. doi: 10.1371/journal.pone.0069124 (PMC3728291; doi:10.1371/journal.pone.0069124)
Supplement: Figure S1 — Sequence and schematic representation of the LuWD40-1 protein encoded by cDNA clone LuP1225D10. (A) Nucleotide and deduced amino acid sequence of LuWD40-1 with 5′ and 3′ untranslated regions. The second in frame ATG selected in the ab initio annotation of Lus10011938 is italicized. The 5′ region amplified for semi-quantitative RT-PCR experiment is underlined (B) Schematic representation of the genomic clone corresponding to LuWD40-1 ORF drawn to scale. The boxes represent exons and lines represent introns. (C) DNA sequence of the genomic clone representing the open reading frame of LuWD40-1. (PDF) [file pone.0069124.s001.pdf]

A

GTCAAAGGGTTTTCAAAATTCGAGAAAATAACTATGAAAGTAATAAGCTAACTTACTGGTATGGTATGTATCGAAAGGCAAAATCCCAAGTTTGAGA
GATTAGGGTTTTTGTCTTTTGGTTTTTTGTTCGACGAACTGCAGATGAAGCAATGAAACCTAAAATTAGTCTACTATTTATACATGAAGGGATAAAC
AAATTAAGGAATGCAAGAATAAAAAACTTATCTCGATCCGGGCAAGAAATCCTTTGCATGCACGATTCGAACTGGATTTGTGTGGATTAAGGCATTT
TTGTCGCTGAAAGGGTTGATTACGGGTAAGTGATGATGATATTGATGATGGATTTGAAGATAATCGATAGAAAGGGAGATTTTGGCGGAAAATTAGG
GTTTGAGTGCTGAATCCTTTGCATGCACGATTCGAACTGGATTTGTGTGGATTGAGACATTTTTGTAGCTGGATACGGGTAAGTGATGATGATATTG
ATGATGGATTAGAAGATAATCGACATAAAAGGAAGATTTTGGCGGAAAATTAGGGTTTGAGTGGCGCTGAATCGAGAGGAAGTCGGCAGAAATTGAG
AGAAAAATCGAGAGGAGGCGTGAAAATTTGGCAGAAATGAAATCCTAATCTGAAAATTTAGCTTTTATACCCTATCAATACTACGTCGTTTTAACTC
GCGGATTGGTTGTGGGTCGGATATGTTGCTATCCGTATCCGCCCATCAACCGAAACGCACGGATCACGGGCCTGACCGAAACCGATAACTATCCGCG
GGCCATGGCTTCCGGGCTGTATTCACGGGCAGGCTCCAATGGGTAATCGAAACCCATGGGCTGCCTGCCAAGCGTACGTCCGATGCCTAAGCCTTGA
ACCTCCACGTTTGCATTTCCGCCTATCGGCGCGACTGCTATTTTCGAATCGAATAAAAGAAGCGGAAGAACAAACTGTAAACTCCATCGTCCCAGGC
TCTGGACTGAAATTCGGAAGGAGGAAACCTCGCTCCAGGTAACGTAAGCTATTTGGACCCCACA

ATGCGC*ATG*GACGCGACGAACTGGGACGAGGTAGCCTATAGAGATAGCATCCTGCGAGAACGAGCAGCCGAAACCCTAAACGTCTTTAGGACGGCA
M R M D A T N W D E V A Y R D S I L R E R A A E T L N V F R T A

TGGGCGCCTTCTTCAGATTTGATCCCTGATGCCATCGTCGTAGCCTCCAGTGACGGGTCGATCAGTTCGTACTCTATATCCTCATGCGCCTCCAAA
W A P S S D L I P D A I V V A S S D G S I S S Y S I S S C A S K

GTTCCGCTGGGTTTCGGCTCTGCAATGGCTCCACAGGTTGCGCCTACTGCCTTTGTTCAAGGCCATGACGGACCAGCATATGATGTCAAATTTTAT
V P L G F G S A M A P Q V A P T A F V Q G H D G P A Y D V K F Y

GGCAGCGGCGAAGATGCCTTATTGTTGAGTTGTGGTGATGATGGTCGGATTCGAGGATGGAAATGGAAAGAATTCATCGAATCTGAGGTTCCAATT
G S G E D A L L L S C G D D G R I R G W K W K E F I E S E V P I

TCATTGCAAGGAAATCATGTGAATTCAGTTGTTGACCTTGTGAATCCCCAACAAAGAGGTCCATGGGGTGCTCTTTCGCCAGTCCCTGAAAACAAT
S L Q G N H V N S V V D L V N P Q Q R G P W G A L S P V P E N N

GCTATTGCTTTAGGCACTAAGGACGCTTCCATCTTTTCTGCTGCTGGTGATTCTTGTGCCTATTGCTGGGATTTGGAGACGTGCAAGATCAAAATG
A I A L G T K D A S I F S A A G D S C A Y C W D L E T C K I K M

GTTTTCAAGGGGCACTTGGATTATTTGCACTGTATAGTTGGCCCCAACTCTGCCAATCAGATTATAACTGGGTCAGAAGATGGAACAGCAAGATTT
V F K G H L D Y L H C I V G P N S A N Q I I T G S E D G T A R F

TGGGATTGCAATAGTGGGAAGTGTGTCAGAGTAATTGACCCAAGGAAGGATAAGAAGTTAAGAAGACCTTCTATGCCTATTAGATGCATTGCACTT
W D C N S G K C V R V I D P R K D K K L R R P S M P I R C I A L

GATGCAAGTGACAACTGGTTGGCTTGTAGCAGCGGTCAAAGTTTATCAGTTTGGAACTTGCATGCTTCTGAGTGTGTTTCGAGGACTTCTACCAGT
D A S D N W L A C S S G Q S L S V W N L H A S E C V S R T S T S

GGTTCCATGCAGGATATAATATTCGACTACAGTCAAGTATTAGCTGTTGGGTCGAAACCTTTTGTCAGTCGATTTGACCTAGATGGAAAGTTTCTA
G S M Q D I I F D Y S Q V L A V G S K P F V S R F D L D G K F L

TCACAAATACCATGTGCTCCTCAGTCAGCATTTTCCATTTCATTACATCCATCGGGGCTGACGGCAATTGGAGGTTATGGGGGTGTTGTAGACGTG
S Q I P C A P Q S A F S I S L H P S G L T A I G G Y G G V V D V

ATATCCCAGGTTCAAAGTCACTATTGCCCATTCCGCTGCAGCCGGGGGTGA

I S Q V Q S H Y C P F R C S R G *

AAGATGTAGATTTGGGTGAGTGAAGGCTCATTTTTGGCTGTGGAATACTCGGAAGTTGGGGGCTGGAGCAGCAGCAGCAGAAACTATACGACATTTT
GATGTCCGTACCAGACTTTGGAAGAAAGGTGTGGAGTTGATGTTTGCAAGTTTAGTTTATACTTGTTTGAGCTGTGTCGCCTCTGCCTCAGCGTCCT
GGCCGTTGTTTGCTCGTTGTTCATGTTGGCGGCTTGCATCGGTGTAAACCAAAATGGTTAACATCAATGGAGCAGGCGAACCAAACTAGAAATTCCC
ATTACAAAACCCCACACGAGTATTTCTCGCTGATGAGGTGAGAGAGCTGACCTTTATTTACACTTTGGATAAACTGACCTTTCTTTATAAAAATAAA
TAACTAGAAGTGAAAAAAAAAAAAAAAAAAAAAAAAAAAAAAAAAAAAAAAAAAAAAAAAG

B

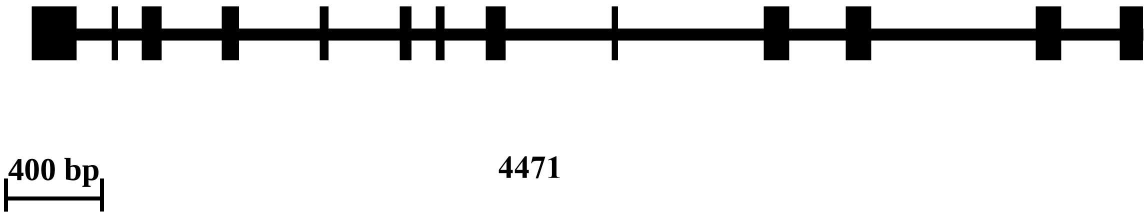

C

ATGCGCATGGACGCGACGAACTGGGACGAGGTAGCCTATAGAGATAGCATCCTGCGAGAACGAGCAGCCGAAACCCTAAACGTCTTTAGGACGGCAT
GGGCGCCTTCTTCAGATTTGATCCCTGATGCCATCGTCGTAGCCTCCAGTGACGGGTCGATCAGTTCGTACTCTATATCCTCATGCGCCTCCAAAGT
TGTAAGTTTCTCTCGCTTCTTTGTTTCTTACAATTTCACTTCAGTCAGCTTCCAATAGAGTAGTTTTGCGTCAGTGACGTGATTGAGTTCCTTTAAC
TTTTTTTTTCTTTTCTGTTGCTCGTTTGGTTTGGCAATCAGCCGCTGGGTTTCGGCTCTGCAATGGCTCCACAGTAAGTCTAAGCGGCTCTGGTGAC
TGTTCCTGACTATAAGCTCCTACTGTCTCTTCTTTGAATTATTCATTACTCTGGTTAATTTACAGGGTTGCGCCTACTGCCTTTGTTCAAGGCCATG
ACGGACCAGCATATGATGTCAAATTTTATGGCAGCGGCGAAGATGCCTTATTGTTGAGGTCTGTTACTATTTTCTTTCTTTTTCATCATTTCCTTTG
ATTTTGAATCCCATCTACATTCGTGCATTTGAATTCGTAGTCGTGTTGGTTTCAGTAAAGCTGGATTGCATCTATTAGTTTGTAGTTTAGTGCACTG
CTGGTGTGCTGGGTGCTGACACGTAATTGCTATGTTGACCCCAATCTGTATTTCTTGTGACGTATGTATATATATTCGATTCCTTGGTATCTGCTTT
ACAGTTGTGGTGATGATGGTCGGATTCGAGGATGGAAATGGAAAGAATTCATCGAATCTGAGGTTCCAATTTCATTGCAAGGTACTAGGCTTAAACG
AAACTGGTATTTTCCTTGTAGCTACATCAACATAGAAACCATTGTCTATTCTCATTTATTGGTACGACTGCTTTTCCATCCACCATGACTCCTGATG
TTAATTTAGAGCGTTATTGGTCTCAGCTTGCTCTTGAAGTTCCTGTTTTTTGTTTTGTTTTGGTATGTATGTCACCTGTTGTTTTATTCGGAGTTAT
GCTTTAGGGGATAGTTTACAATGCTTGTGATTGACTTCTATGATCTTATGTTCCTCTTTCGGCTAAAAGTTGTCTTGCCTTCAAAATCACTGAACAG
GAAATCATGTGAATTCAGTTGTTGACCTTGTGAATCCCCAACAAAGGTGAGCCATAACTCCTGGCATATCTTGACGGATGTTACCTTACTTTTAATT
GATGGCGTTTTCATAAGCAAGCAGGTCGACACAGTATTGGGTTATGGACTATCATGTTTCCATGTTCCCTGGTGGATCAATTACTTTTGACTATAGA
GTGAAATTTCGGTTATTGTGACCTTTGTTTATGGGCCATGGAAGAAGTTAATTTCTTTTGCCATAGGGTTGACATCGTCTAAAACTCTCGCAAATTT
TTAATTGATTTATTTTTGTTTATTGTTGTCTTCAGAGGTCCATGGGGTGCTCTTTCGCCAGTCCCTGAAAACAATGCTATTGCTTTAGGCACTAAGG
TTTGACTCCTATACATAATATATTCCAAGTCCCCCAATTTGAACAAAAAATATATTGTTTAACATTTTGTTGTTCATTAGGACGCTTCCATCTTTTC
TGCTGCTGGTGATTCTTGTGCCTATTGCTGGGATTTGGTATGTATTTGCTCAAGTGCAATTATCGTTGCTATCACATGATACATGCAGCACTATTAC
TTACATATAAGCGTATAGAATGACCATTATGTTTTGTAGCCATTTCAATAACTGGGATTATTATTAATGTTCTACCAACTACTTTTTGTGCTTTCCA
GGAGACGTGCAAGATCAAAATGGTTTTCAAGGGGCACTTGGATTATTTGCACTGTATAGTTGGCCCCAACTCTGCCAATCAGGTAATATTCATTGCT
CTTCACCATTATTCTATTGGTGTAATTAACATCAAGCTTTTCTTAAAATGGTGAGATTGTCCCAAAGGAAATTCGATTTAAGGTGGATTGTTAAATT
GCTGCATAGAAAACTTTCTAGGCAAGTACTCTCTTAATTCTGGGTACACGAGATCCACAAAAGTTTTCAGGTAGTCATCAGTTAGAAAAAAAATTAC
GCCGTTAGCGAATGTTGTATCATTTGATTTGTTGCATGAAATTCTGCCAAGATAATGTGATCAATGCTATAAAACATTTACACTGTTCCGATAGATT
TTTCATTCAATTTCCAATATCAGCCTTATTTATGATAATCTATTTAGCCGCTTATTATTTGTTGTAGACAGTGAACCTGATTGTCCCCCGTTTTCCC
TTACTCTCAGATTATAACTGGGTCAGAAGATGGAACAGCAAGATTTTGGGGTAAATACTGCTTTTGAATATTGACAATCCATGCTTAAAATCAAGAG
AATGTGCCTCACTTGTTTTGAATGTGTTAGTTCATAAATAAAATTGCTGTTAGAGGCAATTGGTGGGAAATTCTTCAATTGTGTGTTTTTCATGTTC
ATTGATCTGGTCCAAGACCCCCATAAGTCATGCAGTTAGGGGATACAATTAGAATGGTCATTAGTTTGGGCGACTGGATTATTTACTTCTTAATTTG
GTATTATTTGGATATTTTTAACAGTTTTAGTTTCTCCAGATCAGATTATAAGATCGTCTTCTTTGGCTTCCTTGTGAGGTATAAATATTGCTTTGCA
GTTGCCCCGGGTTTCCTCCTTTCCCATGAGTACGGGGAAGGCATTTGTGGATGTGGCTGAGCAATGTCTTTCCTTGTTTTTGTGATGGTTTGTTATA
GCAATACTATTGGCAATGTGAATGCCAATTATGGCTTTAAAGCCGACCTTTACCAGTGTGTTTGATGTTCAGTCATGCCATTGCCTGGATTTTATTA
TTCTCTACTACTTTTTGTCTGATGAAATAGAATTGGCGTTTTTAGATTGCAATAGTGGGAAGTGTGTCAGAGTAATTGACCCAAGGAAGGATAAGAA
GTTAAGAAGACCTTCTATGCCTATTAGATGCATTGCACTTGATGCAAGTGACAACTGGTTGGTAAGTGTTTTTTGCTGACAGATTGTACAAGTTAGA
AAAGTGTGATCTTCTACCTTAGATAGGTCTCTTGTATATATGAGAATTAACCGTGTCATCAATAACATAACGAAAAAGTTGGCACTCGTAGTTAGGA
AGAGGAGCTGATACATTCCTCTGCCCTAGCCTGTACTGTGGTTAATGGTGGCCTTATAAATGCATTTTCTATGTTCATAGGCTTGTAGCAGCGGTCA
AAGTTTATCAGTTTGGAACTTGCATGCTTCTGAGTGTGTTTCGAGGACTTCTACCAGTGGTTCCATGCAGGATATAATATTCGACTACAGTCAAGTA
AGGCAATTCTTCCCTAGTTGACCTCCAAATAATCATATGCAGCTTAATTGACCAGGTATCAAAATGGAATTTGCATACCATTTATTAATATATTCTT
TTCAGTAGTTAGGGTATAAGTATAACTGTATAATATCTTTCATTTTCACAAAGCACCTTGGTTGTGGAGGTGCTGGGTTTGGGGATTTGCTTATGAT

TGCAGTTGAAAGTACAAATCCAATTTGTAGGATCCAGAATCAGAATTTGGAATTTTGAACTTGAAGCATGCATTACATTTCACCTTTCCTTGAGTCG
ATTACACAAATAGACAAACTGACGGCTGATCATGAGTTGATGTGTATTTTTAACTATCTGGGAATTATCGTGCTAACCTTATGCCAATCAGGTTAGC
TGTACCTTTCCATTTCTTCAACCATGGTTATTTCGATGATCTCATGTTTCGTGTCCCATGGTTTAACTTAGACACCGGTGATTGATACACCCGTTCA
TTGAACGTATGATTGGTACTGCAGTGGACACCCTTTAGGTGGCGTGTTAAAACCTAGTGTCTTTCCTGTGTTTGACTCTTTTTTGCCATTTTCTTAA
CATTTCTTTCTTTGAGCGGGAGTTGCTGCGTTTGATGACCTCTAAAAATTGTTGCATTATGCAATCTCAGGTATTAGCTGTTGGGTCGAAACCTTTT
GTCAGTCGATTTGACCTAGATGGAAAGTTTCTATCACAAATACCATGTGCTCCTCAGTCAGCATTCTCCATCTCATTACATCCATCGGGGGTATTCT
CTTTTACTTTGACCGAATCTTGGCGTTTCTTGTTCTGTGTTTTGGTAGCTGACATTTCATATATAACATAATCTACCAGAAGTCGTCGTGTTACTTT
TCTATTGTCGAACAAATAATGTGAACTTAAGGGAAGAAAAGAGATATATTGTATACCAATTAGTAATTTGTTTCTAATGTATTGAAATCTGACATTA
ATTTTTTGGGGTACAGCTGACGGCAATTGGAGGTTATGGTGGTGTTGTAGACGTGATATCCCAGGTTCAAAGTCACTATTGCACATTCCGCTGCAGC
CGGGGGTGA

Figure S1 Sequence and schematic representation of the LuWD40-1 protein encoded by cDNA clone LuP1225D10. (A) Nucleotide and deduced amino acid sequence of *LuWD40-1* with 5’ and 3’ untranslated regions. The second in frame ATG selected in the *ab initio* annotation of *Lus10011938* is italicized. The 5’ region amplified for semi-quantitative RT-PCR experiment is underlined (B) Schematic representation of the genomic clone corresponding to *LuWD40-1* ORF drawn to scale. The boxes represent exons and lines represent introns. (C) DNA sequence of the genomic clone representing the open reading frame of *LuWD40-1*
