# Supplementary material for: The LuWD40-1 Gene Encoding WD Repeat Protein Regulates Growth and Pollen Viability in Flax (Linum Usitatissimum L.)
Source: PLoS One. 2013 Jul 30;8(7):e69124. doi: 10.1371/journal.pone.0069124 (PMC3728291; doi:10.1371/journal.pone.0069124)
Supplement: Figure S2 — Semi-quantitative RT-PCR. Amplification of the target gene LuWD40-1 and the control gene apt1 from the three transgenic lines overexpressing LuWD40-1 and the untransformed Prairie Grande shows that the reactions had not reached saturation at 28 cycles but that saturation was achieved at 31 cycles. Ratios (LuWD40-1:apt1) at the bottom were calculated by measuring the amplicon intensity by densitometry using the AlphaImagerHP software version 3.4 (proteinsimple, Santa Clara, CA, USA). Note: the Gateway recombination tags in the LuWD40-1 primers increase the amplification size to 332 bp (274+29 bp tags in each forward and reverse primers). Molecular marker (M) is the 1KB Plus DNA ladder (Invitrogen, Carlsbad, California, USA). (PDF) [file pone.0069124.s002.pdf]

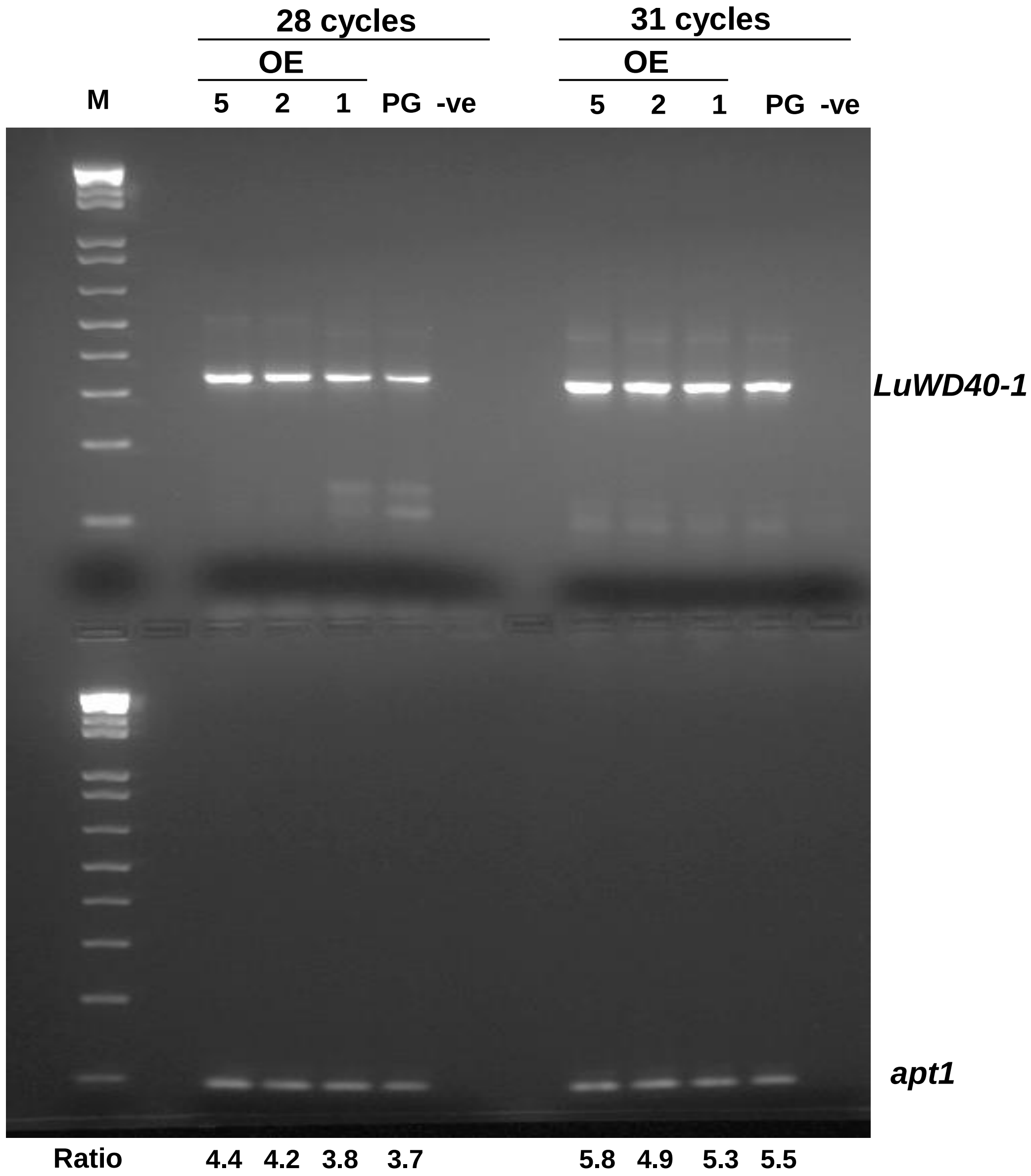

Figure S2 Semi-quantitative RT-PCR. Amplification of the target gene *LuWD40-1* and the control gene *apt1* from the three transgenic lines overexpressing *LuWD40-1* and the untransformed Prairie Grande shows that the reactions had not reached saturation at 28 cycles but that saturation was achieved at 31 cycles. Ratios (*LuWD40-1*:*apt1*) at the bottom were calculated by measuring the amplicon intensity by densitometry using the AlphaImagerHP software version 3.4 (proteinsimple, Santa Clara, CA, USA). Note: the Gateway recombination tags in the *LuWD40-1* primers increase the amplification size to 332bp (274+29bp tags in each forward and reverse primers). Molecular marker (M) is the 1KB Plus DNA ladder (Invitrogen, Carlsbad, California, USA).
