# Supplementary material for: The LuWD40-1 Gene Encoding WD Repeat Protein Regulates Growth and Pollen Viability in Flax (Linum Usitatissimum L.)
Source: PLoS One. 2013 Jul 30;8(7):e69124. doi: 10.1371/journal.pone.0069124 (PMC3728291; doi:10.1371/journal.pone.0069124)
Supplement: Figure S3 — Confirmation of T1 transgenic lines by PCR using CaMV35S and gene specific primer combination on genomic DNA extracted from leaves. Details of primers and PCR conditions are available in the Materials and Methods section. (PDF) [file pone.0069124.s003.pdf]

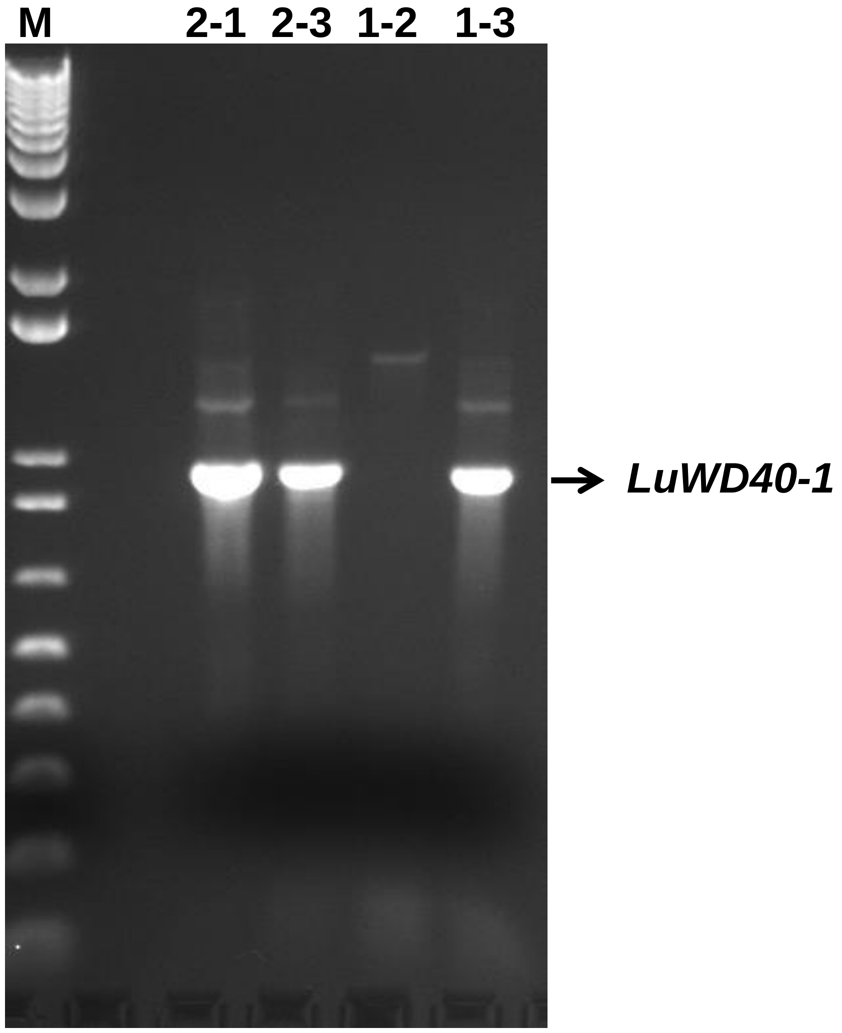

Figure S3 Confirmation of T_1 transgenic lines by PCR using CaMV35S and gene specific primer combination on genomic DNA extracted from leaves. Details of primers and PCR conditions are available in the Materials and Methods section
