# Supplementary material for: The LuWD40-1 Gene Encoding WD Repeat Protein Regulates Growth and Pollen Viability in Flax (Linum Usitatissimum L.)
Source: PLoS One. 2013 Jul 30;8(7):e69124. doi: 10.1371/journal.pone.0069124 (PMC3728291; doi:10.1371/journal.pone.0069124)
Supplement: Figure S4 — Physiological characterization of three LuWD40-1 transgenic lines. Graphs showing differences between Prairie Grande (PG) and overexpressing (OE) lines in physiological parameters: (A) bolting time, (B) branching and (C) height of the plants. (PDF) [file pone.0069124.s004.pdf]

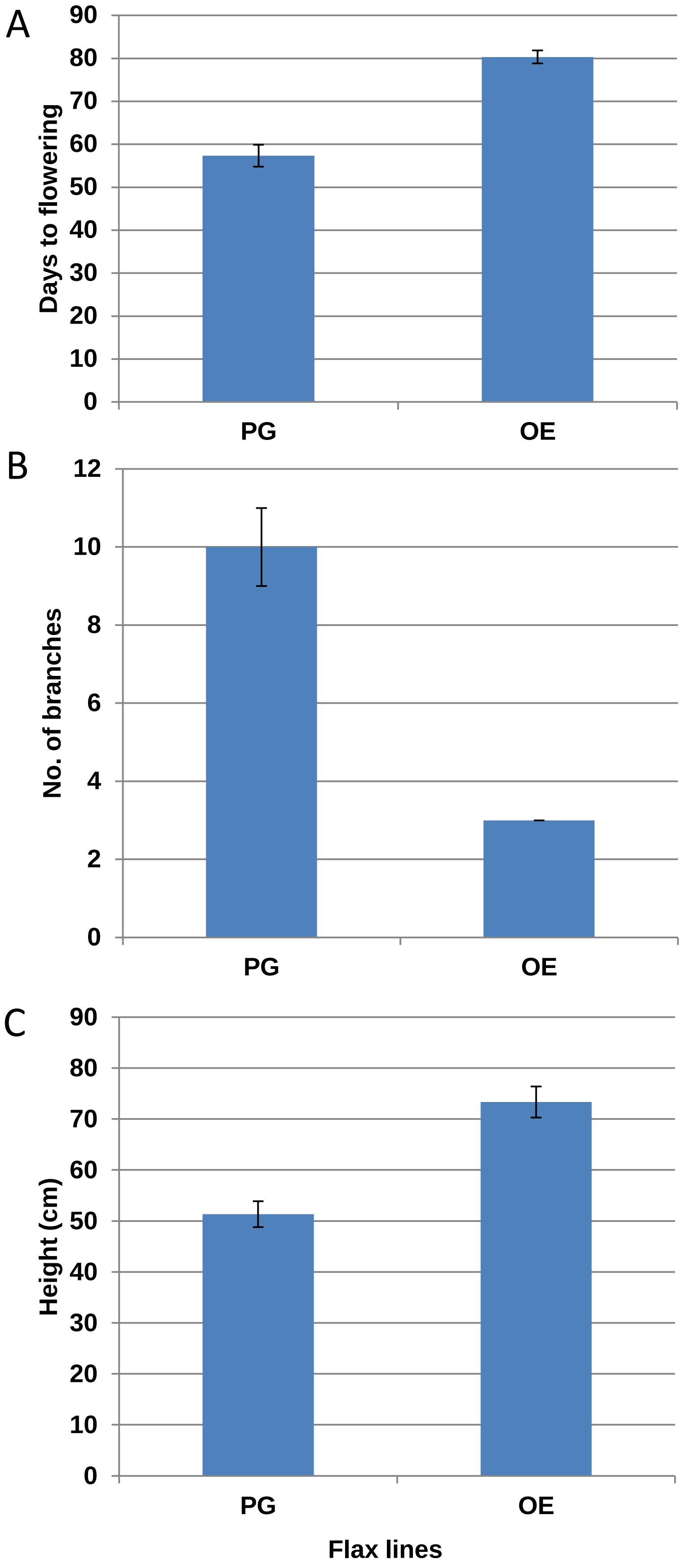

Figure S4 Physiological characterization of three *LuWD40-1* transgenic lines. Graphs showing differences between Prairie Grande (PG) and overexpressing (OE) lines in physiological parameters: (A) bolting time, (B) branching and (C) height of the plants
