# Supplementary material for: The LuWD40-1 Gene Encoding WD Repeat Protein Regulates Growth and Pollen Viability in Flax (Linum Usitatissimum L.)
Source: PLoS One. 2013 Jul 30;8(7):e69124. doi: 10.1371/journal.pone.0069124 (PMC3728291; doi:10.1371/journal.pone.0069124)
Supplement: Table S1 — Transcription factor binding sites in the putative LuWD40-1 promoter. (PDF) [file pone.0069124.s005.pdf]

Table S1 Transcription factor binding sites in the putative *LuWD40-1* promoter.
Sequence Length=1558bp

| Factor | Site | Strand | Seq | Species | Source |
|---|---|---|---|---|---|
| AGL3 | 403 | - | agtgtgccaTATGGatt | Arabidopsis | TRANSFAC |
| AGL3 | 405 | + | tgtgCCATAtgggatttg | Arabidopsis | TRANSFAC |
| AGL3 | 902 | + | aatcCCATAacaatcagt | Arabidopsis | TRANSFAC |
| AGL3 | 1383 | + | caaaCCATAtacttaaac | Arabidopsis | TRANSFAC |
| AG | 134 | + | aggaCCAAAaggagggc | Arabidopsis | TRANSFAC |
| AG | 1378 | + | attaCCAAAccatatact | Arabidopsis | TRANSFAC |
| ANT | 188 | - | agTCGGGtaggaag | Arabidopsis | TRANSFAC |
| Athb-1 | 151 | - | ccagATAATagggg | Arabidopsis | TRANSFAC |
| Athb-1 | 214 | - | cccaATAATgcaac | Arabidopsis | TRANSFAC |
| Athb-1 | 317 | - | atcaATAATctaat | Arabidopsis | TRANSFAC |
| Athb-1 | 1030 | + | gacagATTATtaag | Arabidopsis | TRANSFAC |
| ATHB-5 | 216 | - | cAATAAtgc | Arabidopsis | TRANSFAC |
| ATHB-5 | 319 | - | cAATAAtct | Arabidopsis | TRANSFAC |
| ATHB-5 | 578 | + | ttgTTATTc | Arabidopsis | TRANSFAC |
| ATHB-5 | 779 | + | agtTTATTa | Arabidopsis | TRANSFAC |
| ATHB-5 | 952 | + | ttgTTATTt | Arabidopsis | TRANSFAC |
| ATHB-5 | 1033 | + | agaTTATTa | Arabidopsis | TRANSFAC |
| ATHB-5 | 1294 | + | attTTATTa | Arabidopsis | TRANSFAC |
| ATHB-9 | 322 | + | taatctaATGATtgataac | Arabidopsis | TRANSFAC |
| ATHB-9 | 350 | + | ggtatgaATGATgttacgg | Arabidopsis | TRANSFAC |
| ATHB-9 | 516 | - | gggcaacATCATtttcagg | Arabidopsis | TRANSFAC |
| ATHB-9 | 885 | + | tcatgtaATGATgattaaa | Arabidopsis | TRANSFAC |
| ATHB-9 | 888 | + | tgtaatgATGATtaaatcc | Arabidopsis | TRANSFAC |
| CDC5 | 1178 | + | accTCAGCagt | Arabidopsis | TRANSFAC |
| PIF3 | 69 | + | gattagCACGTtcattag | Arabidopsis | TRANSFAC |
| RAV1 | 45 | + | attCAACAtagc | Arabidopsis | TRANSFAC |
| RAV1 | 516 | + | gggCAACAtcat | Arabidopsis | TRANSFAC |
| RAV1 | 1198 | - | agacTGTTGtca | Arabidopsis | TRANSFAC |
| RAV1 | 1218 | + | gatCAACAccaa | Arabidopsis | TRANSFAC |
| RAV1 | 1224 | + | cacCAACAaaat | Arabidopsis | TRANSFAC |
| RAV1 | 1338 | + | aaaCAACAacca | Arabidopsis | TRANSFAC |
| RAV1 | 1481 | + | acaCAACAttca | Arabidopsis | TRANSFAC |
| GAmyb | 13 | + | CAACCaat | Barley | TRANSFAC |
| GAmyb | 530 | - | tcaGGTTG | Barley | TRANSFAC |
| GAmyb | 1344 | + | CAACCaca | Barley | TRANSFAC |
| GAmyb | 1360 | - | gcaGGTTG | Barley | TRANSFAC |
| Dof1 | 750 | + | agaTAAAGgat | Maize | TRANSFAC |

| | | | | | |
|---|---|---|---|---|---|
| Dof1 | 935 | - | caaCTTTAcat | Maize | TRANSFAC |
| Dof1 | 1079 | - | taaCTTTAaca | Maize | TRANSFAC |
| Dof2 | 35 | + | ggtcAAAGCca | Maize | TRANSFAC |
| Dof2 | 96 | + | tttcAAAGCta | Maize | TRANSFAC |
| Dof2 | 429 | - | agGCTTTcaaa | Maize | TRANSFAC |
| Dof2 | 640 | + | caaaAAAGCat | Maize | TRANSFAC |
| Dof2 | 685 | + | caaaAAAGCag | Maize | TRANSFAC |
| Dof3 | 35 | + | ggtcAAAGCca | Maize | TRANSFAC |
| Dof3 | 96 | + | tttcAAAGCta | Maize | TRANSFAC |
| Dof3 | 429 | - | agGCTTTcaaa | Maize | TRANSFAC |
| Dof3 | 640 | + | caaaAAAGCat | Maize | TRANSFAC |
| Dof3 | 685 | + | caaaAAAGCag | Maize | TRANSFAC |
| O2 | 80 | + | TCATTagttttctaaa | Maize | TRANSFAC |
| O2 | 299 | - | aaacatgtggaAATGA | Maize | TRANSFAC |
| O2 | 317 | - | atcaataatctAATGA | Maize | TRANSFAC |
| O2 | 345 | - | atattggtatgAATGA | Maize | TRANSFAC |
| O2 | 437 | - | aaaaatccatgAATGA | Maize | TRANSFAC |
| O2 | 524 | + | TCATTttcaggttggt | Maize | TRANSFAC |
| O2 | 672 | + | TCATTgaagaagacaa | Maize | TRANSFAC |
| O2 | 717 | + | TCATTgaagaaggtag | Maize | TRANSFAC |
| O2 | 808 | + | TCATTttcatatttga | Maize | TRANSFAC |
| O2 | 880 | - | gtcactcatgtAATGA | Maize | TRANSFAC |
| PBF | 138 | + | ccaAAAAGgag | Maize | TRANSFAC |
| PBF | 397 | + | aagAAAAGtgt | Maize | TRANSFAC |
| PBF | 640 | + | caaAAAAGcat | Maize | TRANSFAC |
| PBF | 685 | + | caaAAAAGcag | Maize | TRANSFAC |
| PBF | 798 | + | tttAAAAGatt | Maize | TRANSFAC |
| PBF | 919 | + | tcaAAAAGgat | Maize | TRANSFAC |
| P | 191 | - | cgGGTAGga | Maize | TRANSFAC |
| P | 726 | - | aaGGTAGat | Maize | TRANSFAC |
| MYB.Ph3 | 78 | + | gttcatTAGTTtt | Petunia | TRANSFAC |
| MYB.Ph3 | 489 | + | agggctTAGTTga | Petunia | TRANSFAC |
| MYB.Ph3 | 780 | + | gtttatTAGTTta | Petunia | TRANSFAC |
| MYB.Ph3 | 961 | + | cagaatTAGTTag | Petunia | TRANSFAC |
| MYB.Ph3 | 1087 | + | acaattTAGTTca | Petunia | TRANSFAC |
| MYB.Ph3 | 1259 | - | ccAACTAtttgac | Petunia | TRANSFAC |
| LIM1 | 559 | - | agggagtGGTGG | Tobacco | TRANSFAC |
| LIM1 | 562 | - | gagtggtGGTGG | Tobacco | TRANSFAC |
| TEIL | 239 | + | ATGTAaat | Tobacco | TRANSFAC |
| TEIL | 664 | - | agtTACAT | Tobacco | TRANSFAC |
| TEIL | 709 | - | agtTACAT | Tobacco | TRANSFAC |
| TEIL | 770 | + | ATGTAtaa | Tobacco | TRANSFAC |
| TEIL | 887 | + | ATGTAatg | Tobacco | TRANSFAC |
| TEIL | 938 | - | cttTACAT | Tobacco | TRANSFAC |
| TEIL | 1298 | - | tatTACAT | Tobacco | TRANSFAC |
| TEIL | 1399 | - | actTACAT | Tobacco | TRANSFAC |
| -10PEHVPSBD | 593 | + | TATTCT | Barley | PLACE |

| | | | | | |
|---|---|---|---|---|---|
| -300CORE | 956 | - | CTTTACA | maize/wheat/barley/tobacco | PLACE |
| -300CORE | 956 | - | CTTTACA | maize/wheat/barley/tobacco | PLACE |
| -300CORE | 956 | - | CTTTACA | maize/wheat/barley/tobacco | PLACE |
| -300CORE | 956 | - | CTTTACA | maize/wheat/barley/tobacco | PLACE |
| -300ELEMENT | 955 | - | ACTTTACA | wheat | PLACE |
| ABI4 | 1266 | + | CTGCCTCC | Maize | JASPER |
| ABI4 | 1383 | - | AGGAGCAG | Maize | JASPER |
| ABI4 | 1522 | - | AAGAGCAG | Maize | JASPER |
| ABI4 | 520 | - | GAGGGCGG | Maize | JASPER |
| ABI4 | 701 | - | AAAAGCAG | Maize | JASPER |
| ABRELATERD1 | 76 | - | CACGT | Arabidopsis | PLACE |
| ABRERATCAL | 75 | - | GCACGTT | Arabidopsis | PLACE |
| ACGTATERD1 | 77 | + | ACGT | Arabidopsis | PLACE |
| ACGTATERD1 | 77 | - | ACGT | Arabidopsis | PLACE |
| ANAERO1CONSENSUS | 287 | - | TTTGTTT | maize/Arabidopsis/pea/barley/rice | PLACE |
| ANAERO1CONSENSUS | 287 | - | TTTGTTT | maize/Arabidopsis/pea/barley/rice | PLACE |
| ANAERO1CONSENSUS | 287 | - | TTTGTTT | maize/Arabidopsis/pea/barley/rice | PLACE |
| ANAERO1CONSENSUS | 287 | - | TTTGTTT | maize/Arabidopsis/pea/barley/rice | PLACE |
| ANAERO1CONSENSUS | 287 | - | TTTGTTT | maize/Arabidopsis/pea/barley/rice | PLACE |
| AP1 | 140 | - | CCAAAAA | Arabidopsis | AGRIS |
| AP1 | 178 | - | CTAAAAA | Arabidopsis | AGRIS |
| ARR10 | 1053 | + | AGATTATT | Arabidopsis | JASPER |
| ARR10 | 156 | + | AGATAATA | Arabidopsis | JASPER |
| ARR10 | 326 | - | AATAATCT | Arabidopsis | JASPER |
| ARR10 | 378 | + | AGATACTA | Arabidopsis | JASPER |
| ARR10 | 69 | + | AGATTAGC | Arabidopsis | JASPER |
| ARR10 | 888 | + | AGATTTGA | Arabidopsis | JASPER |
| ARR1AT | 1053 | + | AGATT | Arabidopsis | PLACE |
| ARR1AT | 1132 | - | AATCT | Arabidopsis | PLACE |
| ARR1AT | 124 | + | CGATT | Arabidopsis | PLACE |
| ARR1AT | 1276 | - | AATCA | Arabidopsis | PLACE |
| ARR1AT | 1353 | - | AATCC | Arabidopsis | PLACE |
| ARR1AT | 1444 | - | AATCA | Arabidopsis | PLACE |

| | | | | | |
|---|---|---|---|---|---|
| ARR1AT | 1489 | - | AATCT | Arabidopsis | PLACE |
| ARR1AT | 18 | - | AATCA | Arabidopsis | PLACE |
| ARR1AT | 22 | - | AATCA | Arabidopsis | PLACE |
| ARR1AT | 329 | - | AATCT | Arabidopsis | PLACE |
| ARR1AT | 336 | + | TGATT | Arabidopsis | PLACE |
| ARR1AT | 424 | + | GGATT | Arabidopsis | PLACE |
| ARR1AT | 448 | - | AATCC | Arabidopsis | PLACE |
| ARR1AT | 490 | + | TGATT | Arabidopsis | PLACE |
| ARR1AT | 555 | + | GGATT | Arabidopsis | PLACE |
| ARR1AT | 635 | + | TGATT | Arabidopsis | PLACE |
| ARR1AT | 69 | + | AGATT | Arabidopsis | PLACE |
| ARR1AT | 751 | + | AGATT | Arabidopsis | PLACE |
| ARR1AT | 772 | + | GGATT | Arabidopsis | PLACE |
| ARR1AT | 779 | + | AGATT | Arabidopsis | PLACE |
| ARR1AT | 820 | + | AGATT | Arabidopsis | PLACE |
| ARR1AT | 846 | + | GGATT | Arabidopsis | PLACE |
| ARR1AT | 888 | + | AGATT | Arabidopsis | PLACE |
| ARR1AT | 913 | + | TGATT | Arabidopsis | PLACE |
| ARR1AT | 920 | - | AATCC | Arabidopsis | PLACE |
| ARR1AT | 931 | - | AATCA | Arabidopsis | PLACE |
| ARR1AT | 944 | + | GGATT | Arabidopsis | PLACE |
| AtMYC2 | 114 | - | CATGTG | Arabidopsis | AGRIS |
| AtMYC2 | 308 | - | CATGTG | Arabidopsis | AGRIS |
| Athb-1 | 334 | - | AATGATTG | Arabidopsis | JASPER |
| BIHD1OS | 1229 | + | TGTCA | rice | PLACE |
| BIHD1OS | 628 | + | TGTCA | rice | PLACE |
| BIHD1OS | 896 | + | TGTCA | rice | PLACE |
| BOXIINTPATPB | 1468 | - | TTCTAT | tobacco | PLACE |
| BOXLCOREDCPAL | 543 | - | GGTTGGT | carrot | PLACE |
| C1MOTIFZMBZ2 | 1114 | - | TAGTTCA | maize | PLACE |
| C1MOTIFZMBZ2 | 1284 | + | CCAACTA | maize | PLACE |
| C1MOTIFZMBZ2 | 1410 | + | CAAACCA | maize | PLACE |
| C1MOTIFZMBZ2 | 801 | - | TAGTTTA | maize | PLACE |
| C1MOTIFZMBZ2 | 986 | - | TAGTTAG | maize | PLACE |
| CAATBOX1 | 1014 | + | CAAT | pea | PLACE |
| CAATBOX1 | 1016 | - | ATTG | pea | PLACE |
| CAATBOX1 | 1109 | + | CAAT | pea | PLACE |
| CAATBOX1 | 1131 | + | CAAT | pea | PLACE |
| CAATBOX1 | 1189 | + | CAAT | pea | PLACE |
| CAATBOX1 | 1279 | + | CAAT | pea | PLACE |
| CAATBOX1 | 1344 | - | ATTG | pea | PLACE |
| CAATBOX1 | 1378 | - | ATTG | pea | PLACE |
| CAATBOX1 | 1403 | + | CAAT | pea | PLACE |
| CAATBOX1 | 1452 | + | CAAT | pea | PLACE |
| CAATBOX1 | 17 | + | CAAT | pea | PLACE |
| CAATBOX1 | 21 | + | CAAT | pea | PLACE |
| CAATBOX1 | 220 | + | CAAT | pea | PLACE |

| | | | | | |
|---|---|---|---|---|---|
| CAATBOX1 | 325 | + | CAAT | pea | PLACE |
| CAATBOX1 | 338 | - | ATTG | pea | PLACE |
| CAATBOX1 | 353 | - | ATTG | pea | PLACE |
| CAATBOX1 | 395 | - | ATTG | pea | PLACE |
| CAATBOX1 | 588 | - | ATTG | pea | PLACE |
| CAATBOX1 | 637 | - | ATTG | pea | PLACE |
| CAATBOX1 | 687 | - | ATTG | pea | PLACE |
| CAATBOX1 | 733 | - | ATTG | pea | PLACE |
| CAATBOX1 | 855 | - | ATTG | pea | PLACE |
| CAATBOX1 | 860 | - | ATTG | pea | PLACE |
| CAATBOX1 | 930 | + | CAAT | pea | PLACE |
| CAATBOX1 | 970 | - | ATTG | pea | PLACE |
| CATATGGMSAUR | 418 | + | CATATG | soybean | PLACE |
| CATATGGMSAUR | 418 | - | CATATG | soybean | PLACE |
| CATATGGMSAUR | 624 | + | CATATG | soybean | PLACE |
| CATATGGMSAUR | 624 | - | CATATG | soybean | PLACE |
| CCA1ATLHCB1 | 1129 | + | AACAATCT | Arabidopsis | PLACE |
| CCA1 | 1129 | + | AACAATCT | Arabidopsis | AGRIS |
| CCAATBOX1 | 1451 | + | CCAAT | Soybean | PLACE |
| CCAATBOX1 | 16 | + | CCAAT | Soybean | PLACE |
| CCAATBOX1 | 219 | + | CCAAT | Soybean | PLACE |
| CCAATBOX1 | 860 | - | ATTGG | Soybean | PLACE |
| CEREGLUBOX1PSLEGA | 1102 | - | ACTTTAACA | pea | PLACE |
| CIACADIANLELHC | 885 | - | GATAGATTTG | tomato | PLACE |
| CPBCSPOR | 798 | + | TATTAG | cucumber | PLACE |
| Core | 1055 | + | ATTA | Arabidopsis | AGRIS |
| Core | 1058 | + | ATTA | Arabidopsis | AGRIS |
| Core | 1088 | - | TAAT | Arabidopsis | AGRIS |
| Core | 1090 | + | ATTA | Arabidopsis | AGRIS |
| Core | 1140 | + | ATTA | Arabidopsis | AGRIS |
| Core | 1330 | + | ATTA | Arabidopsis | AGRIS |
| Core | 1405 | + | ATTA | Arabidopsis | AGRIS |
| Core | 1465 | - | TAAT | Arabidopsis | AGRIS |
| Core | 1488 | - | TAAT | Arabidopsis | AGRIS |
| Core | 159 | - | TAAT | Arabidopsis | AGRIS |
| Core | 223 | - | TAAT | Arabidopsis | AGRIS |
| Core | 328 | - | TAAT | Arabidopsis | AGRIS |
| Core | 333 | - | TAAT | Arabidopsis | AGRIS |
| Core | 557 | + | ATTA | Arabidopsis | AGRIS |
| Core | 66 | + | ATTA | Arabidopsis | AGRIS |
| Core | 71 | + | ATTA | Arabidopsis | AGRIS |
| Core | 753 | + | ATTA | Arabidopsis | AGRIS |
| Core | 774 | + | ATTA | Arabidopsis | AGRIS |
| Core | 799 | + | ATTA | Arabidopsis | AGRIS |
| Core | 83 | + | ATTA | Arabidopsis | AGRIS |
| Core | 872 | + | ATTA | Arabidopsis | AGRIS |
| Core | 907 | - | TAAT | Arabidopsis | AGRIS |

| | | | | | |
|---|---|---|---|---|---|
| Core | 984 | + | ATTA | Arabidopsis | AGRIS |
| DOFCOREZM | 1044 | + | AAAG | maize | PLACE |
| DOFCOREZM | 1076 | + | AAAG | maize | PLACE |
| DOFCOREZM | 1103 | - | CTTT | maize | PLACE |
| DOFCOREZM | 144 | + | AAAG | maize | PLACE |
| DOFCOREZM | 1521 | + | AAAG | maize | PLACE |
| DOFCOREZM | 286 | - | CTTT | maize | PLACE |
| DOFCOREZM | 39 | + | AAAG | maize | PLACE |
| DOFCOREZM | 409 | + | AAAG | maize | PLACE |
| DOFCOREZM | 440 | - | CTTT | maize | PLACE |
| DOFCOREZM | 6 | + | AAAG | maize | PLACE |
| DOFCOREZM | 656 | + | AAAG | maize | PLACE |
| DOFCOREZM | 702 | + | AAAG | maize | PLACE |
| DOFCOREZM | 769 | + | AAAG | maize | PLACE |
| DOFCOREZM | 818 | + | AAAG | maize | PLACE |
| DOFCOREZM | 941 | + | AAAG | maize | PLACE |
| DOFCOREZM | 956 | - | CTTT | maize | PLACE |
| DPBFCOREDCDC3 | 114 | - | CATGTGT | carrot/Arabidopsis | PLACE |
| DPBFCOREDCDC3 | 1437 | + | ACACTGG | carrot/Arabidopsis | PLACE |
| Dof2 | 39 | + | AAAGCC | Maize | JASPER |
| Dof2 | 438 | - | GGCTTT | Maize | JASPER |
| Dof2 | 656 | + | AAAGCA | Maize | JASPER |
| Dof2 | 702 | + | AAAGCA | Maize | JASPER |
| Dof3 | 1044 | + | AAAGTA | Maize | JASPER |
| Dof3 | 1076 | + | AAAGTA | Maize | JASPER |
| Dof3 | 39 | + | AAAGCC | Maize | JASPER |
| Dof3 | 409 | + | AAAGTG | Maize | JASPER |
| Dof3 | 438 | - | GGCTTT | Maize | JASPER |
| Dof3 | 656 | + | AAAGCA | Maize | JASPER |
| Dof3 | 702 | + | AAAGCA | Maize | JASPER |
| EBOXBNNAPA | 1014 | + | CAATTG | rape | PLACE |
| EBOXBNNAPA | 1014 | - | CAATTG | rape | PLACE |
| EBOXBNNAPA | 114 | + | CATGTG | rape | PLACE |
| EBOXBNNAPA | 114 | - | CATGTG | rape | PLACE |
| EBOXBNNAPA | 308 | + | CATGTG | rape | PLACE |
| EBOXBNNAPA | 308 | - | CATGTG | rape | PLACE |
| EBOXBNNAPA | 418 | + | CATATG | rape | PLACE |
| EBOXBNNAPA | 418 | - | CATATG | rape | PLACE |
| EBOXBNNAPA | 486 | + | CATCTG | rape | PLACE |
| EBOXBNNAPA | 486 | - | CATCTG | rape | PLACE |
| EBOXBNNAPA | 599 | + | CAACTG | rape | PLACE |
| EBOXBNNAPA | 599 | - | CAACTG | rape | PLACE |
| EBOXBNNAPA | 624 | + | CATATG | rape | PLACE |
| EBOXBNNAPA | 624 | - | CATATG | rape | PLACE |
| EBOXBNNAPA | 631 | + | CATCTG | rape | PLACE |

| | | | | | |
|---|---|---|---|---|---|
| EBOXBNNAPA | 631 | - | CATCTG | rape | PLACE |
| ELRECOREPCRP1 | 35 | - | GGTCAA | parsley/tobacco | PLACE |
| EMHVCHORD | 955 | - | ACTTTACA | barley | PLACE |
| ERELEE4 | 848 | + | ATTTCAAA | tomato/carnation /Lycopersicon | PLACE |
| ERF1 | 476 | - | GGCGGC | Arabidopsis | AGRIS |
| GATABOX | 1038 | - | TATC | petunia/Arabidopsis/rice | PLACE |
| GATABOX | 1038 | - | TATC | petunia/Arabidopsis/rice | PLACE |
| GATABOX | 1038 | - | TATC | petunia/Arabidopsis/rice | PLACE |
| GATABOX | 157 | + | GATA | petunia/Arabidopsis/rice | PLACE |
| GATABOX | 157 | + | GATA | petunia/Arabidopsis/rice | PLACE |
| GATABOX | 157 | + | GATA | petunia/Arabidopsis/rice | PLACE |
| GATABOX | 250 | - | TATC | petunia/Arabidopsis/rice | PLACE |
| GATABOX | 250 | - | TATC | petunia/Arabidopsis/rice | PLACE |
| GATABOX | 250 | - | TATC | petunia/Arabidopsis/rice | PLACE |
| GATABOX | 322 | - | TATC | petunia/Arabidopsis/rice | PLACE |
| GATABOX | 322 | - | TATC | petunia/Arabidopsis/rice | PLACE |
| GATABOX | 322 | - | TATC | petunia/Arabidopsis/rice | PLACE |
| GATABOX | 341 | + | GATA | petunia/Arabidopsis/rice | PLACE |
| GATABOX | 341 | + | GATA | petunia/Arabidopsis/rice | PLACE |
| GATABOX | 341 | + | GATA | petunia/Arabidopsis/rice | PLACE |
| GATABOX | 379 | + | GATA | petunia/Arabidopsis/rice | PLACE |
| GATABOX | 379 | + | GATA | petunia/Arabidopsis/rice | PLACE |
| GATABOX | 379 | + | GATA | petunia/Arabidopsis/rice | PLACE |
| GATABOX | 708 | + | GATA | petunia/Arabidopsis/rice | PLACE |
| GATABOX | 708 | + | GATA | petunia/Arabidopsis/rice | PLACE |

| | | | | | |
|---|---|---|---|---|---|
| GATABOX | 708 | + | GATA | petunia/Arabidopsis/rice | PLACE |
| GATABOX | 746 | + | GATA | petunia/Arabidopsis/rice | PLACE |
| GATABOX | 746 | + | GATA | petunia/Arabidopsis/rice | PLACE |
| GATABOX | 746 | + | GATA | petunia/Arabidopsis/rice | PLACE |
| GATABOX | 761 | + | GATA | petunia/Arabidopsis/rice | PLACE |
| GATABOX | 761 | + | GATA | petunia/Arabidopsis/rice | PLACE |
| GATABOX | 761 | + | GATA | petunia/Arabidopsis/rice | PLACE |
| GATABOX | 766 | + | GATA | petunia/Arabidopsis/rice | PLACE |
| GATABOX | 766 | + | GATA | petunia/Arabidopsis/rice | PLACE |
| GATABOX | 766 | + | GATA | petunia/Arabidopsis/rice | PLACE |
| GATABOX | 877 | + | GATA | petunia/Arabidopsis/rice | PLACE |
| GATABOX | 877 | + | GATA | petunia/Arabidopsis/rice | PLACE |
| GATABOX | 877 | + | GATA | petunia/Arabidopsis/rice | PLACE |
| GATABOX | 885 | + | GATA | petunia/Arabidopsis/rice | PLACE |
| GATABOX | 885 | + | GATA | petunia/Arabidopsis/rice | PLACE |
| GATABOX | 885 | + | GATA | petunia/Arabidopsis/rice | PLACE |
| GBF5 | 1007 | - | ATGAGT | Arabidopsis | AGRIS |
| GBF5 | 900 | + | ACTCAT | Arabidopsis | AGRIS |
| GCCCORE | 476 | - | GGCGGC | Arabidopsis/tomato | PLACE |
| GCCCORE | 476 | - | GGCGGC | Arabidopsis/tomato | PLACE |
| GT1CONSENSUS | 1405 | - | ATTACC | pea/oat/rice/tobacco/Arabidopsis | PLACE |
| GT1CONSENSUS | 1405 | - | ATTACC | pea/oat/rice/tobacco/Arabidopsis | PLACE |
| GT1CONSENSUS | 1405 | - | ATTACC | pea/oat/rice/tobacco/Arabidopsis | PLACE |

| | | | | | |
|---|---|---|---|---|---|
| GT1CONSENSUS | 1405 | - | ATTACC | pea/oat/rice/tobacco/Arabidopsis | PLACE |
| GT1CONSENSUS | 157 | + | GATAAT | pea/oat/rice/tobacco/Arabidopsis | PLACE |
| GT1CONSENSUS | 157 | + | GATAAT | pea/oat/rice/tobacco/Arabidopsis | PLACE |
| GT1CONSENSUS | 157 | + | GATAAT | pea/oat/rice/tobacco/Arabidopsis | PLACE |
| GT1CONSENSUS | 157 | + | GATAAT | pea/oat/rice/tobacco/Arabidopsis | PLACE |
| GT1CONSENSUS | 313 | + | GGAAAT | pea/oat/rice/tobacco/Arabidopsis | PLACE |
| GT1CONSENSUS | 313 | + | GGAAAT | pea/oat/rice/tobacco/Arabidopsis | PLACE |
| GT1CONSENSUS | 313 | + | GGAAAT | pea/oat/rice/tobacco/Arabidopsis | PLACE |
| GT1CONSENSUS | 313 | + | GGAAAT | pea/oat/rice/tobacco/Arabidopsis | PLACE |
| GT1CONSENSUS | 390 | + | GGAAAA | pea/oat/rice/tobacco/Arabidopsis | PLACE |
| GT1CONSENSUS | 390 | + | GGAAAA | pea/oat/rice/tobacco/Arabidopsis | PLACE |
| GT1CONSENSUS | 390 | + | GGAAAA | pea/oat/rice/tobacco/Arabidopsis | PLACE |
| GT1CONSENSUS | 390 | + | GGAAAA | pea/oat/rice/tobacco/Arabidopsis | PLACE |
| GT1CONSENSUS | 391 | + | GAAAAT | pea/oat/rice/tobacco/Arabidopsis | PLACE |
| GT1CONSENSUS | 391 | + | GAAAAT | pea/oat/rice/tobacco/Arabidopsis | PLACE |

| | | | | | |
|---|---|---|---|---|---|
| GT1CONSENSUS | 391 | + | GAAAAT | pea/oat/rice/tobacco/Arabidopsis | PLACE |
| GT1CONSENSUS | 391 | + | GAAAAT | pea/oat/rice/tobacco/Arabidopsis | PLACE |
| GT1CONSENSUS | 536 | - | ATTTTC | pea/oat/rice/tobacco/Arabidopsis | PLACE |
| GT1CONSENSUS | 536 | - | ATTTTC | pea/oat/rice/tobacco/Arabidopsis | PLACE |
| GT1CONSENSUS | 536 | - | ATTTTC | pea/oat/rice/tobacco/Arabidopsis | PLACE |
| GT1CONSENSUS | 536 | - | ATTTTC | pea/oat/rice/tobacco/Arabidopsis | PLACE |
| GT1CONSENSUS | 619 | - | TTTTTC | pea/oat/rice/tobacco/Arabidopsis | PLACE |
| GT1CONSENSUS | 619 | - | TTTTTC | pea/oat/rice/tobacco/Arabidopsis | PLACE |
| GT1CONSENSUS | 619 | - | TTTTTC | pea/oat/rice/tobacco/Arabidopsis | PLACE |
| GT1CONSENSUS | 619 | - | TTTTTC | pea/oat/rice/tobacco/Arabidopsis | PLACE |
| GT1CONSENSUS | 766 | + | GATAAA | pea/oat/rice/tobacco/Arabidopsis | PLACE |
| GT1CONSENSUS | 766 | + | GATAAA | pea/oat/rice/tobacco/Arabidopsis | PLACE |
| GT1CONSENSUS | 766 | + | GATAAA | pea/oat/rice/tobacco/Arabidopsis | PLACE |
| GT1CONSENSUS | 766 | + | GATAAA | pea/oat/rice/tobacco/Arabidopsis | PLACE |
| GT1CONSENSUS | 826 | - | ATTTTC | pea/oat/rice/tobacco/Arabidopsis | PLACE |

| | | | | | |
|---|---|---|---|---|---|
| GT1CONSENSUS | 826 | - | ATTTTC | pea/oat/rice/tobacco/Arabidopsis | PLACE |
| GT1CONSENSUS | 826 | - | ATTTTC | pea/oat/rice/tobacco/Arabidopsis | PLACE |
| GT1CONSENSUS | 826 | - | ATTTTC | pea/oat/rice/tobacco/Arabidopsis | PLACE |
| GT1CORE | 840 | + | GGTTAA | pea | PLACE |
| GT1GMSCAM4 | 619 | - | TTTTTC | soybean | PLACE |
| HMG-1 | 1444 | - | AATCACACC | Pea | JASPER |
| HMG-1 | 644 | - | AAACAAGAC | Pea | JASPER |
| HMG-1 | 690 | - | GAAGAAGAC | Pea | JASPER |
| HMG-IY | 282 | - | GTTTCTTTGTTTGTGC | Pea | JASPER |
| HMG-IY | 689 | + | TGAAGAAGACAAAAAA | Pea | JASPER |
| HMG-IY | 892 | - | TTGATGTCACTCATGT | Pea | JASPER |
| INRNTPSADB | 314 | - | GAAATGAA | tobacco | PLACE |
| INRNTPSADB | 393 | - | AAATTGAA | tobacco | PLACE |
| INRNTPSADB | 823 | + | TTCATTTT | tobacco | PLACE |
| INRNTPSADB | 853 | - | AAATTGAA | tobacco | PLACE |
| LTRECOREATCOR15 | 192 | - | GTCGG | Arabidopsis/rape | PLACE |
| LTRECOREATCOR15 | 192 | - | GTCGG | Arabidopsis/rape | PLACE |
| MNB1A | 1044 | + | AAAGT | Maize | JASPER |
| MNB1A | 1076 | + | AAAGT | Maize | JASPER |
| MNB1A | 1102 | - | ACTTT | Maize | JASPER |
| MNB1A | 1521 | + | AAAGA | Maize | JASPER |
| MNB1A | 285 | - | TCTTT | Maize | JASPER |
| MNB1A | 39 | + | AAAGC | Maize | JASPER |
| MNB1A | 409 | + | AAAGT | Maize | JASPER |
| MNB1A | 439 | - | GCTTT | Maize | JASPER |
| MNB1A | 6 | + | AAAGA | Maize | JASPER |
| MNB1A | 656 | + | AAAGC | Maize | JASPER |
| MNB1A | 702 | + | AAAGC | Maize | JASPER |
| MNB1A | 818 | + | AAAGA | Maize | JASPER |
| MNB1A | 955 | - | ACTTT | Maize | JASPER |
| MYB.ph3 | 1027 | + | TAACAGTTT | Petunia | JASPER |
| MYB.ph3 | 1027 | - | TAACAGTTT | Petunia | JASPER |
| MYB.ph3 | 1081 | - | AGACAGTTA | Petunia | JASPER |
| MYB.ph3 | 1106 | + | TAACAATTT | Petunia | JASPER |
| MYB.ph3 | 1128 | + | TAACAATCT | Petunia | JASPER |
| MYB.ph3 | 1161 | + | TAACAGTCT | Petunia | JASPER |
| MYB.ph3 | 1178 | + | TAACTGTTA | Petunia | JASPER |

| | | | | | |
|---|---|---|---|---|---|
| MYB.ph3 | 1178 | - | TAACTGTTA | Petunia | JASPER |
| MYB.ph3 | 673 | + | TAACAGTTA | Petunia | JASPER |
| MYB.ph3 | 673 | - | TAACAGTTA | Petunia | JASPER |
| MYB.ph3 | 719 | + | TAACAGTTA | Petunia | JASPER |
| MYB.ph3 | 719 | - | TAACAGTTA | Petunia | JASPER |
| MYB.ph3 | 790 | + | TAACAGTTT | Petunia | JASPER |
| MYB.ph3 | 790 | - | TAACAGTTT | Petunia | JASPER |
| MYB.ph3 | 798 | - | TATTAGTTT | Petunia | JASPER |
| MYB.ph3 | 927 | + | TAACAATCA | Petunia | JASPER |
| MYB.ph3 | 983 | - | AATTAGTTA | Petunia | JASPER |
| MYB1AT | 1411 | + | AAACCA | Arabidopsis | PLACE |
| MYB1AT | 546 | - | TGGTTT | Arabidopsis | PLACE |
| MYB2AT | 1084 | - | CAGTTA | Arabidopsis | PLACE |
| MYB2AT | 1178 | + | TAACTG | Arabidopsis | PLACE |
| MYB2AT | 271 | + | TAACTG | Arabidopsis | PLACE |
| MYB2AT | 676 | - | CAGTTA | Arabidopsis | PLACE |
| MYB2AT | 722 | - | CAGTTA | Arabidopsis | PLACE |
| MYB2CONSENSUSAT | 1084 | - | CAGTTA | Arabidopsis | PLACE |
| MYB2CONSENSUSAT | 1178 | + | TAACTG | Arabidopsis | PLACE |
| MYB2CONSENSUSAT | 271 | + | TAACTG | Arabidopsis | PLACE |
| MYB2CONSENSUSAT | 29 | + | CAACGG | Arabidopsis | PLACE |
| MYB2CONSENSUSAT | 599 | + | CAACTG | Arabidopsis | PLACE |
| MYB2CONSENSUSAT | 676 | - | CAGTTA | Arabidopsis | PLACE |
| MYB2CONSENSUSAT | 722 | - | CAGTTA | Arabidopsis | PLACE |
| MYB4 | 1368 | + | AACAACC | Arabidopsis | AGRIS |
| MYB4 | 1408 | + | ACCAAAC | Arabidopsis | AGRIS |
| MYB4 | 543 | - | GGTTGGT | Arabidopsis | AGRIS |
| MYBCOREATCYCB1 | 30 | + | AACGG | Arabidopsis | PLACE |
| MYBCORE | 1027 | - | TAACAG | Arabidopsis/petunia | PLACE |
| MYBCORE | 1027 | - | TAACAG | Arabidopsis/petunia | PLACE |
| MYBCORE | 1084 | + | CAGTTA | Arabidopsis/petunia | PLACE |
| MYBCORE | 1084 | + | CAGTTA | Arabidopsis/petunia | PLACE |
| MYBCORE | 1161 | - | TAACAG | Arabidopsis/petunia | PLACE |
| MYBCORE | 1161 | - | TAACAG | Arabidopsis/petunia | PLACE |
| MYBCORE | 1178 | - | TAACTG | Arabidopsis/petunia | PLACE |
| MYBCORE | 1178 | - | TAACTG | Arabidopsis/petunia | PLACE |
| MYBCORE | 1181 | + | CTGTTA | Arabidopsis/petunia | PLACE |

| | | | | | |
|---|---|---|---|---|---|
| MYBCORE | 1181 | + | CTGTTA | Arabidopsis/petunia | PLACE |
| MYBCORE | 1225 | + | CTGTTG | Arabidopsis/petunia | PLACE |
| MYBCORE | 1225 | + | CTGTTG | Arabidopsis/petunia | PLACE |
| MYBCORE | 1502 | - | TAACAG | Arabidopsis/petunia | PLACE |
| MYBCORE | 1502 | - | TAACAG | Arabidopsis/petunia | PLACE |
| MYBCORE | 271 | - | TAACTG | Arabidopsis/petunia | PLACE |
| MYBCORE | 271 | - | TAACTG | Arabidopsis/petunia | PLACE |
| MYBCORE | 29 | - | CAACGG | Arabidopsis/petunia | PLACE |
| MYBCORE | 29 | - | CAACGG | Arabidopsis/petunia | PLACE |
| MYBCORE | 599 | - | CAACTG | Arabidopsis/petunia | PLACE |
| MYBCORE | 599 | - | CAACTG | Arabidopsis/petunia | PLACE |
| MYBCORE | 673 | - | TAACAG | Arabidopsis/petunia | PLACE |
| MYBCORE | 673 | - | TAACAG | Arabidopsis/petunia | PLACE |
| MYBCORE | 676 | + | CAGTTA | Arabidopsis/petunia | PLACE |
| MYBCORE | 676 | + | CAGTTA | Arabidopsis/petunia | PLACE |
| MYBCORE | 719 | - | TAACAG | Arabidopsis/petunia | PLACE |
| MYBCORE | 719 | - | TAACAG | Arabidopsis/petunia | PLACE |
| MYBCORE | 722 | + | CAGTTA | Arabidopsis/petunia | PLACE |
| MYBCORE | 722 | + | CAGTTA | Arabidopsis/petunia | PLACE |
| MYBCORE | 790 | - | TAACAG | Arabidopsis/petunia | PLACE |
| MYBCORE | 790 | - | TAACAG | Arabidopsis/petunia | PLACE |
| MYBPLANT | 543 | - | GGTTGGTT | snapdragon/bean/petunia/Arabidopsis | PLACE |

| | | | | | |
|---|---|---|---|---|---|
| MYBPLANT | 543 | - | GGTTGGTT | snapdragon/bean/petunia/Arabidopsis | PLACE |
| MYBPLANT | 543 | - | GGTTGGTT | snapdragon/bean/petunia/Arabidopsis | PLACE |
| MYBPLANT | 543 | - | GGTTGGTT | snapdragon/bean/petunia/Arabidopsis | PLACE |
| MYBPZM | 196 | - | GGTAGG | maize | PLACE |
| MYBPZM | 543 | - | GGTTGG | maize | PLACE |
| MYCATERD1 | 114 | + | CATGTG | Arabidopsis | PLACE |
| MYCATERD1 | 308 | + | CATGTG | Arabidopsis | PLACE |
| MYCATRD22 | 114 | - | CATGTG | Arabidopsis | PLACE |
| MYCATRD22 | 308 | - | CATGTG | Arabidopsis | PLACE |
| MYCCONSENSUSAT | 1014 | + | CAATTG | Arabidopsis | PLACE |
| MYCCONSENSUSAT | 1014 | - | CAATTG | Arabidopsis | PLACE |
| MYCCONSENSUSAT | 114 | + | CATGTG | Arabidopsis | PLACE |
| MYCCONSENSUSAT | 114 | - | CATGTG | Arabidopsis | PLACE |
| MYCCONSENSUSAT | 308 | + | CATGTG | Arabidopsis | PLACE |
| MYCCONSENSUSAT | 308 | - | CATGTG | Arabidopsis | PLACE |
| MYCCONSENSUSAT | 418 | + | CATATG | Arabidopsis | PLACE |
| MYCCONSENSUSAT | 418 | - | CATATG | Arabidopsis | PLACE |
| MYCCONSENSUSAT | 486 | + | CATCTG | Arabidopsis | PLACE |
| MYCCONSENSUSAT | 486 | - | CATCTG | Arabidopsis | PLACE |
| MYCCONSENSUSAT | 599 | + | CAACTG | Arabidopsis | PLACE |
| MYCCONSENSUSAT | 599 | - | CAACTG | Arabidopsis | PLACE |
| MYCCONSENSUSAT | 624 | + | CATATG | Arabidopsis | PLACE |
| MYCCONSENSUSAT | 624 | - | CATATG | Arabidopsis | PLACE |
| MYCCONSENSUSAT | 631 | + | CATCTG | Arabidopsis | PLACE |
| MYCCONSENSUSAT | 631 | - | CATCTG | Arabidopsis | PLACE |
| NAPINMOTIFBN | 115 | - | ATGTGTA | rape | PLACE |
| NODCON1GM | 818 | + | AAAGAT | soybean | PLACE |
| NODCON2GM | 1522 | - | AAGAG | soybean | PLACE |
| NODCON2GM | 172 | + | CTCTT | soybean | PLACE |
| NODCON2GM | 666 | - | AAGAG | soybean | PLACE |
| OSE1ROOTNODULE | 818 | + | AAAGAT | bean/Medicago/soybean/Sesbania | PLACE |
| OSE1ROOTNODULE | 818 | + | AAAGAT | bean/Medicago/soybean/Sesbania | PLACE |
| OSE1ROOTNODULE | 818 | + | AAAGAT | bean/Medicago/soybean/Sesbania | PLACE |

| | | | | | |
|---|---|---|---|---|---|
| OSE2ROOTNODULE | 1522 | - | AAGAG | bean/Medicago/soybean/Sesbania | PLACE |
| OSE2ROOTNODULE | 1522 | - | AAGAG | bean/Medicago/soybean/Sesbania | PLACE |
| OSE2ROOTNODULE | 1522 | - | AAGAG | bean/Medicago/soybean/Sesbania | PLACE |
| OSE2ROOTNODULE | 172 | + | CTCTT | bean/Medicago/soybean/Sesbania | PLACE |
| OSE2ROOTNODULE | 172 | + | CTCTT | bean/Medicago/soybean/Sesbania | PLACE |
| OSE2ROOTNODULE | 172 | + | CTCTT | bean/Medicago/soybean/Sesbania | PLACE |
| OSE2ROOTNODULE | 666 | - | AAGAG | bean/Medicago/soybean/Sesbania | PLACE |
| OSE2ROOTNODULE | 666 | - | AAGAG | bean/Medicago/soybean/Sesbania | PLACE |
| OSE2ROOTNODULE | 666 | - | AAGAG | bean/Medicago/soybean/Sesbania | PLACE |
| PBF | 1044 | + | AAAGT | Maize | JASPER |
| PBF | 1076 | + | AAAGT | Maize | JASPER |
| PBF | 1102 | - | ACTTT | Maize | JASPER |
| PBF | 39 | + | AAAGC | Maize | JASPER |
| PBF | 409 | + | AAAGT | Maize | JASPER |
| PBF | 439 | - | GCTTT | Maize | JASPER |
| PBF | 656 | + | AAAGC | Maize | JASPER |
| PBF | 702 | + | AAAGC | Maize | JASPER |
| PBF | 955 | - | ACTTT | Maize | JASPER |
| POLASIG1 | 796 | - | TTTATT | pea/rice/Arabidopsis | PLACE |
| POLASIG1 | 796 | - | TTTATT | pea/rice/Arabidopsis | PLACE |
| POLASIG1 | 796 | - | TTTATT | pea/rice/Arabidopsis | PLACE |
| POLASIG3 | 1055 | - | ATTATT | maize | PLACE |
| POLASIG3 | 221 | + | AATAAT | maize | PLACE |
| POLASIG3 | 326 | + | AATAAT | maize | PLACE |
| POLLEN1LELAT52 | 1042 | + | AGAAA | tomato | PLACE |
| POLLEN1LELAT52 | 1074 | + | AGAAA | tomato | PLACE |

| | | | | | |
|---|---|---|---|---|---|
| POLLEN1LELAT52 | 1215 | + | AGAAA | tomato | PLACE |
| POLLEN1LELAT52 | 1455 | - | TTTCT | tomato | PLACE |
| POLLEN1LELAT52 | 283 | - | TTTCT | tomato | PLACE |
| POLLEN1LELAT52 | 641 | + | AGAAA | tomato | PLACE |
| POLLEN1LELAT52 | 89 | - | TTTCT | tomato | PLACE |
| PREATPRODH | 1007 | - | ATGAGT | Arabidopsis | PLACE |
| PREATPRODH | 900 | + | ACTCAT | Arabidopsis | PLACE |
| PYRIMIDINEBOXOSRAMY1A | 143 | - | AAAAGG | rice/barley | PLACE |
| PYRIMIDINEBOXOSRAMY1A | 143 | - | AAAAGG | rice/barley | PLACE |
| PYRIMIDINEBOXOSRAMY1A | 940 | - | AAAAGG | rice/barley | PLACE |
| PYRIMIDINEBOXOSRAMY1A | 940 | - | AAAAGG | rice/barley | PLACE |
| RAV1-A | 1226 | - | TGTTG | Arabidopsis | AGRIS |
| RAV1-A | 1245 | + | CAACA | Arabidopsis | AGRIS |
| RAV1-A | 1251 | + | CAACA | Arabidopsis | AGRIS |
| RAV1-A | 1367 | + | CAACA | Arabidopsis | AGRIS |
| RAV1-A | 1513 | + | CAACA | Arabidopsis | AGRIS |
| RAV1-A | 529 | + | CAACA | Arabidopsis | AGRIS |
| RAV1AAT | 1226 | - | TGTTG | Arabidopsis | PLACE |
| RAV1AAT | 1245 | + | CAACA | Arabidopsis | PLACE |
| RAV1AAT | 1251 | + | CAACA | Arabidopsis | PLACE |
| RAV1AAT | 1367 | + | CAACA | Arabidopsis | PLACE |
| RAV1AAT | 1513 | + | CAACA | Arabidopsis | PLACE |
| RAV1AAT | 529 | + | CAACA | Arabidopsis | PLACE |
| RBCSCONSENSUS | 1353 | + | AATCCAA | tomato/petunia/tobacco/pea | PLACE |
| RBCSCONSENSUS | 1353 | + | AATCCAA | tomato/petunia/tobacco/pea | PLACE |
| RBCSCONSENSUS | 1353 | + | AATCCAA | tomato/petunia/tobacco/pea | PLACE |
| RBCSCONSENSUS | 1353 | + | AATCCAA | tomato/petunia/tobacco/pea | PLACE |
| REALPHALGLHCB21 | 14 | + | AACCAA | duckweed | PLACE |
| REALPHALGLHCB21 | 545 | - | TTGGTT | duckweed | PLACE |
| RHERPATEXPA7 | 75 | + | GCACGT | Arabidopsis | PLACE |
| RUNX1 | 834 | + | ATTTGAGGTTA | Arabidopsis | JASPER |
| RYREPEATBNNAPA | 295 | - | TGCATG | rape | PLACE |
| RYREPEATBNNAPA | 297 | + | CATGCA | rape | PLACE |
| RYREPEATLEGUMINBOX | 294 | - | GTGCATG | soybean | PLACE |
| SEF4MOTIFGM7S | 179 | - | TAAAAAC | soybean | PLACE |
| SEF4MOTIFGM7S | 444 | - | CAAAAAT | soybean | PLACE |
| SORLIP1AT | 1507 | + | GCCAC | Arabidopsis | PLACE |
| SORLIP2AT | 1460 | - | GGCCC | Arabidopsis | PLACE |
| SP8BFIBSP8AIB | 273 | + | ACTGTGTA | sweet | potato |
| SURECOREATSULTR11 | 167 | + | GAGAC | Arabidopsis | PLACE |
| TAAAGSTKST1 | 1103 | - | CTTTA | potato | PLACE |
| TAAAGSTKST1 | 768 | + | TAAAG | potato | PLACE |
| TAAAGSTKST1 | 956 | - | CTTTA | potato | PLACE |

| | | | | | |
|---|---|---|---|---|---|
| TATABOX5 | 974 | + | TTATTT | pea | PLACE |
| TGBOXATPIN2 | 76 | - | CACGTT | tomato/Arabidopsis | PLACE |
| TGBOXATPIN2 | 76 | - | CACGTT | tomato/Arabidopsis | PLACE |
| WBBOXPCWRKY1 | 1291 | + | TTTGACT | sweet | potato/wheat/barley |
| WBBOXPCWRKY1 | 1291 | + | TTTGACT | sweet | potato/wheat/barley |
| WBBOXPCWRKY1 | 35 | - | GGTCAAA | sweet | potato/wheat/barley |
| WBBOXPCWRKY1 | 35 | - | GGTCAAA | sweet | potato/wheat/barley |
| WBBOXPCWRKY1 | 935 | - | AGTCAAA | sweet | potato/wheat/barley |
| WBBOXPCWRKY1 | 935 | - | AGTCAAA | sweet | potato/wheat/barley |
| WBOXATNPR1 | 1292 | + | TTGAC | Arabidopsis | PLACE |
| WBOXATNPR1 | 36 | - | GTCAA | Arabidopsis | PLACE |
| WBOXATNPR1 | 401 | - | GTCAA | Arabidopsis | PLACE |
| WBOXATNPR1 | 936 | - | GTCAA | Arabidopsis | PLACE |
| WBOXHVISO1 | 1293 | + | TGACT | barley | PLACE |
| WBOXHVISO1 | 400 | - | AGTCA | barley | PLACE |
| WBOXHVISO1 | 935 | - | AGTCA | barley | PLACE |
| WBOXNTERF3 | 1293 | + | TGACT | tobacco | PLACE |
| WBOXNTERF3 | 35 | - | GGTCA | tobacco | PLACE |
| WBOXNTERF3 | 400 | - | AGTCA | tobacco | PLACE |
| WBOXNTERF3 | 935 | - | AGTCA | tobacco | PLACE |
| WRKY71OS | 1230 | - | GTCA | rice/parsley | PLACE |
| WRKY71OS | 1293 | + | TGAC | rice/parsley | PLACE |
| WRKY71OS | 36 | - | GTCA | rice/parsley | PLACE |
| WRKY71OS | 401 | - | GTCA | rice/parsley | PLACE |
| WRKY71OS | 629 | - | GTCA | rice/parsley | PLACE |
| WRKY71OS | 897 | - | GTCA | rice/parsley | PLACE |
| WRKY71OS | 936 | - | GTCA | rice/parsley | PLACE |
| XYLAT | 284 | - | TTCTTTGT | Arabidopsis | PLACE |
| | | | | | |
